# An Efficient Synthesis of Optically Active [4-^13^C] Labelled Quorum Sensing Signal Autoinducer-2

**DOI:** 10.3390/molecules26020369

**Published:** 2021-01-12

**Authors:** Osvaldo S. Ascenso, Gonzalo Carrau, Karina B. Xavier, M. Rita Ventura, Christopher D. Maycock

**Affiliations:** 1 Instituto de Tecnologia Química e Biológica António Xavier, Universidade Nova de Lisboa, 2780-157 Oeiras, Portugal; oascenso@itqb.unl.pt (O.S.A.); gcarrau@itqb.unl.pt (G.C.); 2 Facultad de Química, Universidad de la República, Montevideo 11800, Uruguay; 3 Instituto Gulbenkian de Ciência, 2780-156 Oeiras, Portugal; kxavier@igc.gulbenkian.pt; 4 Faculdade de Ciências da Universidade de Lisboa, Departamento de Química e Bioquímica, 1749-016 Lisboa, Portugal

**Keywords:** DPD, AI-2, quorum sensing, enantioselective synthesis, ^13^C-DPD

## Abstract

A new synthetic route for the quorum sensing signal Autoinducer-2 (AI-2) is described and used for the preparation of [4-^13^C]-AI-2 starting from [1-^13^C]-bromoacetic acid. The key step in this process was the enantioselective reduction of an intermediate ketone. This synthesis provides, selectively, both enantiomers of the labelled or unlabelled parent compound, (*R*) or (*S*)-4,5-dihydroxypentane-2,3-dione (DPD) and was used for an improved synthesis of [1-^13^C]-AI-2.

## 1. Introduction

**Scheme 1:**
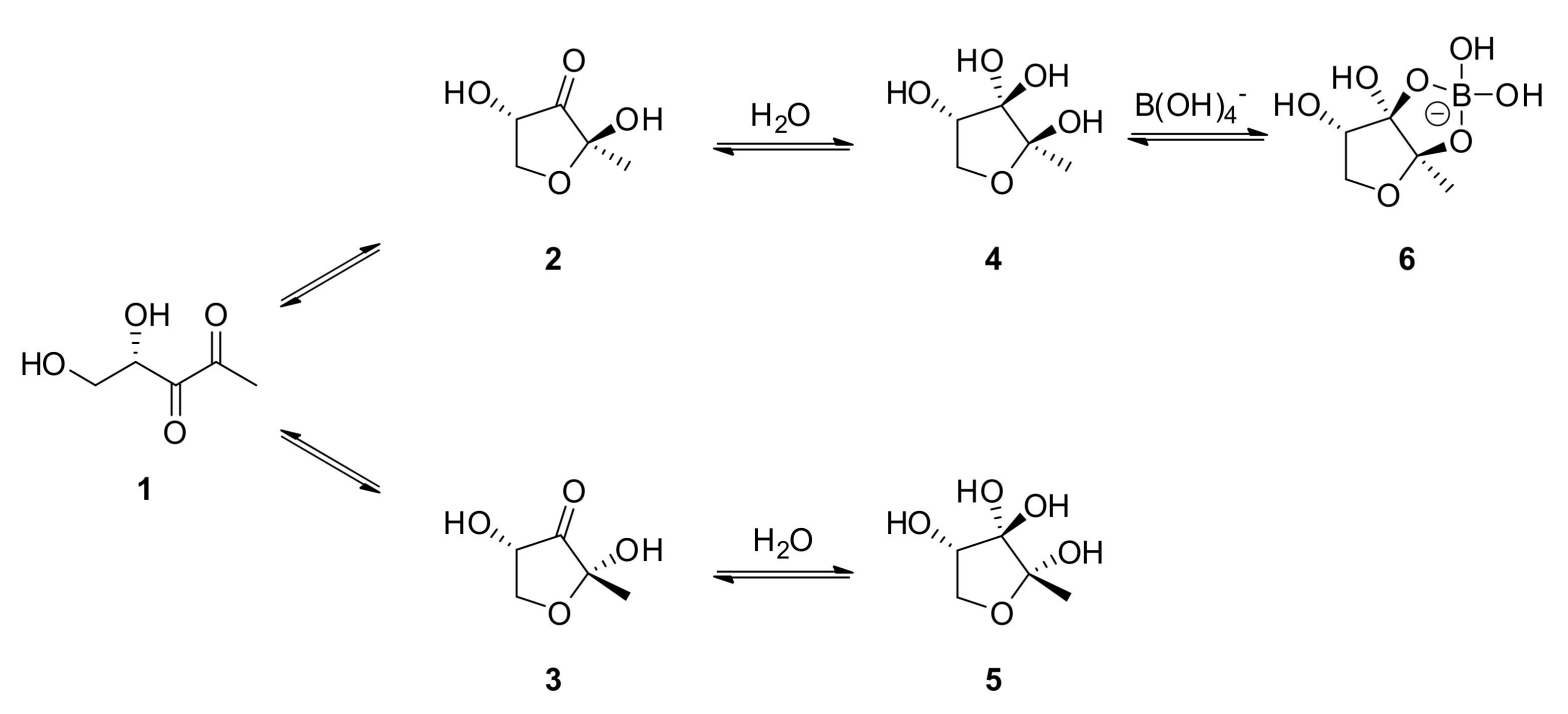
AI-2 is an equilibrium mixture of the linear and cyclic forms of DPD **1**.

Bacterial populations use cell-cell communication to coordinate their behaviour, facilitating adaptation to changing environments. Chemical communication among bacteria is called “quorum sensing”. Examples of quorum sensing-regulated behaviours are biofilm formation, virulence-factor expression, antibiotic production and bioluminescence [1,2]. Autoinducer-2 (AI-2) is produced and detected by many phylogenetically distinct bacteria [3,4]. Using AI-2, bacteria are able to detect the presence of other bacterial species in their vicinity and regulate gene expression according to the species composition in the environment [5,6]. (*S*)-4,5-dihydroxypentane-2,3-dione (DPD) **1** is the precursor of AI-2 that in aqueous medium is an equilibrium mixture of the linear form, two anomeric cyclic forms **2** and **3,** and their hydrated versions **4** and **5** (Scheme 1) [7].

It has been shown that many species of Gram-positive and Gram-negative bacteria can detect different forms of this molecule and thus all these forms are known collectively as AI-2. Specifically, many *Vibrio* have the LuxP-type of AI-2 receptor which recognises borated isomer **6** [8], whereas members belonging to phylogenetically distinct families such as the human pathogens *Salmonella typhimurium* and *Bacillus anthracis*, as well as different *Clostridium* and the plant symbiont *Sinorhizobium meliloti* having the LsrB-type of receptors, recognise the non-borated diastereoisomer **5** [9,10]. Bacteria that have the LsrB-type of receptor internalise this signal via an ABC transporter. Once inside the cell the non-borated form of AI-2 is phosphorylated and in bacteria such as *Escherichia coli* the phosphorylated compound is cleaved by a thiolase (LsrF) into metabolites which are channelled into glycolysis and the citric acid cycle, as means to terminate quorum sensing [11]. This thiolase is not present in all bacteria that have the LsrB receptor, indicating that AI-2 metabolism might not be as conserved as the receptors [10].

Ultimately, the understanding of the molecular mechanisms that bacteria use to regulate their behaviours can lead to the development of new therapies to control bacterial infections and also to develop biotechnological applications for the control of the industrial scale production of beneficial bacterial products, such as antibiotics or recombinant proteins.

Even though naturally occurring (*S*)-DPD (AI-2) is a small molecule, it is highly functionalised, optically active and unstable at high concentrations. This constitutes a challenge for producing significant amounts of this product. To the best of our knowledge, there have been described to date three racemic syntheses [12,13,14], four syntheses of the enantiopure form starting from the chiral pool [7,15,16,17], and our group has previously described the synthesis of the natural enantiomer starting from non-chiral starting materials [18]. This route has proven to be efficient, highly reproducible and can be easily modified to generate new analogues in the optically pure form [19,20].

**Figure 1:**
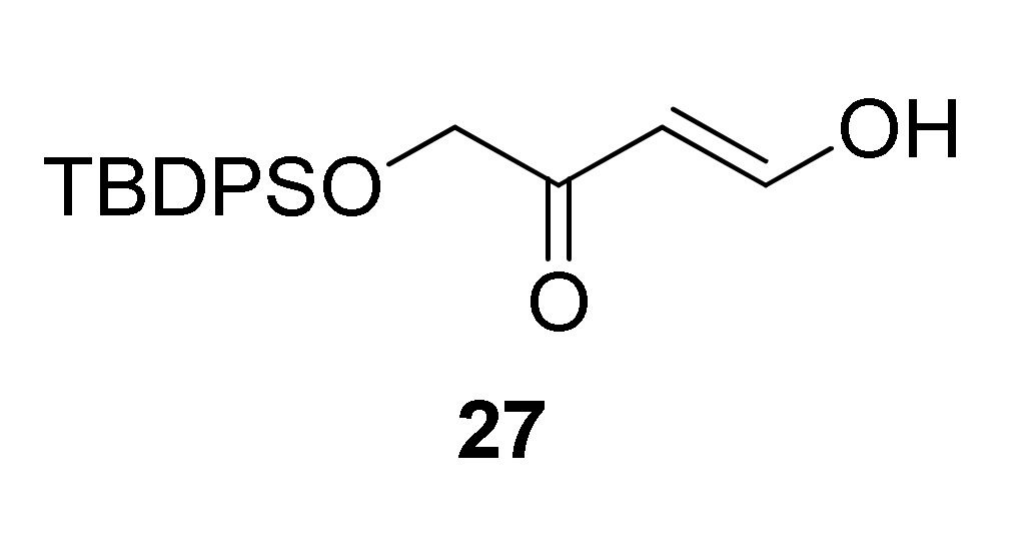
By-product **27** from the reaction of the Weinreb amide **18** with lithium trimethylsilylacetylene (Scheme 3).

The asymmetric synthesis of DPD is fundamental since only one enantiomer is produced and recognised by bacteria. Studies have shown that the unnatural borated (*R*)-DPD showed only 1% of the activity of the borated (*S*)-DPD form **6** (Figure 1) in the *V. harveyi* bioassay (LuxP receptor) [7]. For the assay of the isomer **5** (LsrB receptor), to induce the *E. coli* reporter, six times more (*R*)-DPD than (*S*)-DPD was necessary in order to obtain the same activity [18]. Thus, the obtention of DPD with the highest enantiopurity is important for accurate biological studies.

The direct detection of AI-2 in biological samples is not easy, due to the complexity of the samples, low natural abundance and the instability of AI-2. A liquid chromatography-tandem mass spectrometric method for the quantification of AI-2 in saliva samples has been described [21]. For this, extracted AI-2 was converted into a corresponding more stable quinoxaline before being analysed and [1-^13^C]-DPD was used as the internal standard. To date, this is the only ^13^C labelled DPD reported [7].

This work describes a considerable improvement of our previous synthesis of optically active DPD [18] and has been applied to the synthesis of [4-^13^C]-DPD.

## 2. Results and Discussion

[4-^13^C]-DPD is particularly important for in vivo studies, since it has been shown that AI-2, when internalised in *Escherichia coli*, is processed to dihydroxyacetone phosphate (DHAP) and acetyl-CoA, and these are key metabolites that are then used by the cell into central metabolism via glycolysis and the citric acid cycle [22]. In this final enzymatic reaction, the AI-2 molecule is broken: The C-1 is incorporated into acetyl-CoA and the other carbons remain in DHAP. Thus, [4-^13^C]-DPD is important as it allows the labelling of a different metabolic product of AI-2 than from [1-^13^C]-DPD. The metabolic fate of AI-2 in bacteria that do not have homologues to the LsrF-thiolase, such as several *Clostridium*, has yet to be determined [10]. Since the ^13^C labelled starting material is relatively expensive and the labelled carbon atom is introduced in the first step of the synthesis, it was important to improve the enantioselectivity and global yield of the DPD synthesis and avoid time consuming chromatographic purifications.

**Scheme 2:**
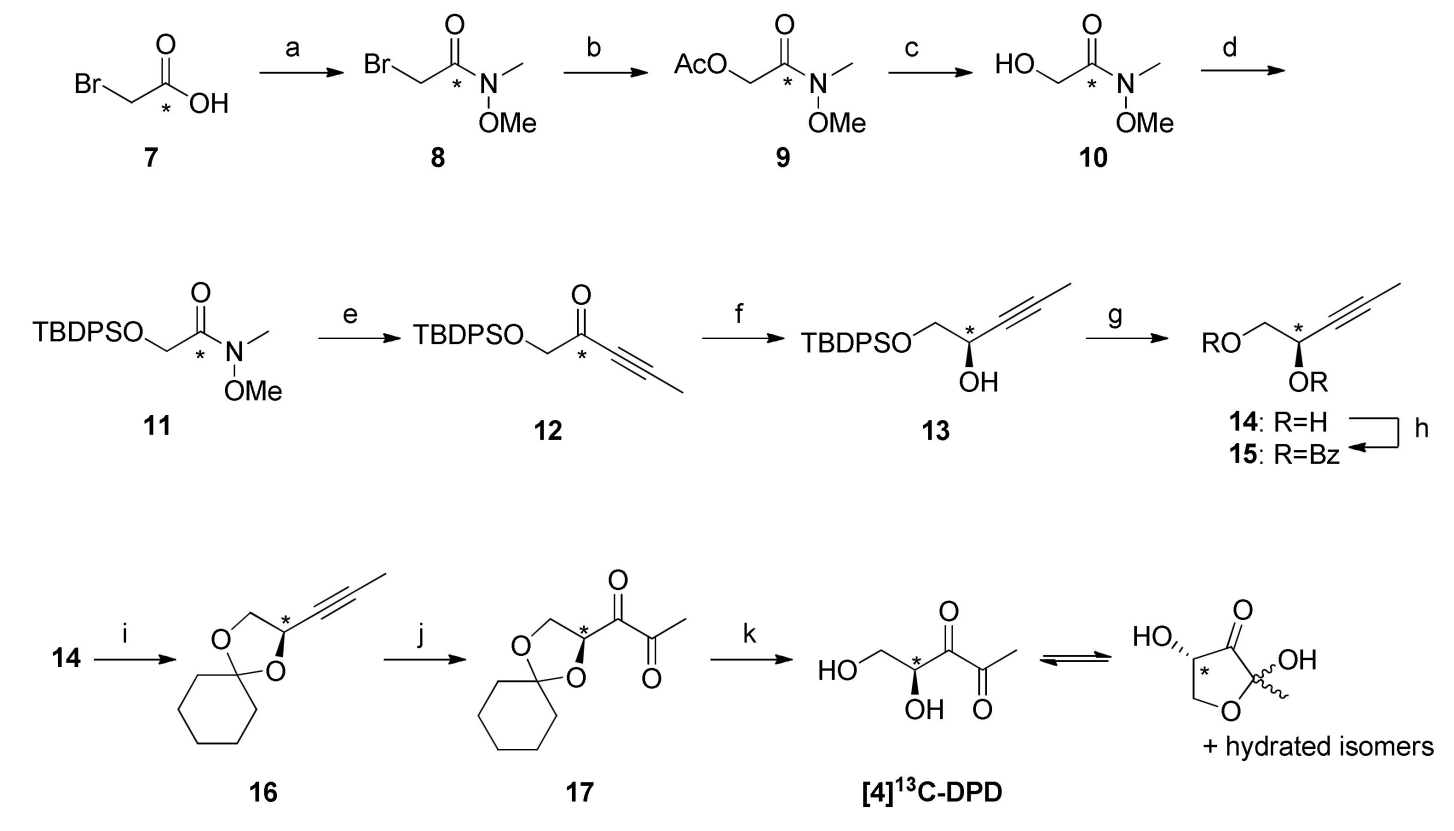
Synthesis of [4-^13^C]-DPD. Reagents and conditions: (**a**) PBr_3_, MeNH(OMe), toluene, from 0 °C to 60 °C, 88%; (**b**) AcOH, Et_3_N, ClCH_2_CH_2_Cl, reflux, 86%; (**c**) MeONa, MeOH, 0 °C, 83%; (**d**) TBDPSCl, Et_3_N, DMAP, CH_2_Cl_2_, from 0 °C to rt, 100%; (**e**) *n*-BuLi, Propyne, THF, −78–0 °C, 95%; (**f**) Ru(*p*-cymene)[(*S,S*)-TsDPEN], *i*-PrOH, rt, 95% (*ee* = 94%); (**g**) TBAF, THF, rt, 86%; (**h**) BzCl, (*i*-Pr)_2_NEt, DMAP, CH_2_Cl_2_, 93%; (**i**) 1,1-dimethoxycyclohexane, H_2_SO_4_, DMF, 91%; (**j**) NaIO_4_, RuO_2_, CCl_4_/MeCN, rt, 86%; (**k**) Dowex 50WX8, H_2_O, pH 3, rt.

Compound **11** was prepared by a four-step sequence starting from [1-^13^C]-bromoacetic acid (**7**), as illustrated in Scheme 2. [1-^13^C]-Bromoacetic acid was chosen over [1-^13^C]-glycolic acid since it was commercially available and less expensive. For the synthesis of the unlabelled DPD, bromoacetic acid is economically the best starting material even though more steps are required. Weinreb amide **8** was formed in 88% yield using a procedure described by Hu and coworkers [23]. Acetolysis of the bromide **8** afforded compound **9** in 86% yield [24] and the acetate group methanolised to obtain alcohol **10** in 83% yield. It is noteworthy that attempts to directly hydrolyse bromide **8** were unsuccessful and the expected alcohol **10** was never isolated from these experiments. Finally, the alcohol was protected with the *tert*-butyldiphenylsilyl (TBDPS) group, rendering compound **11** in 63% overall yield. This synthetic route does not require any chromatographic purification until the last step. From Weinreb amide **11**, an aqueous solution of [4-^13^C]-DPD was obtained following the same synthetic method reported previously (Scheme 2), except that the asymmetric reduction was carried out by the Noyori reduction [25] instead of the Midland reduction described previously [18]. The Noyori procedure afforded alcohol **13** in 95% yield with an *ee* of 94%, thus avoiding the recrystallisation step used to enhance the enantiomeric excess in previous work [18]. Furthermore, the purification of **13** obtained using Noyori’s chiral Ru catalyst was much more straightforward than when using (*S*)-Alpine borane as the chiral reduction agent.

**Scheme 3:**
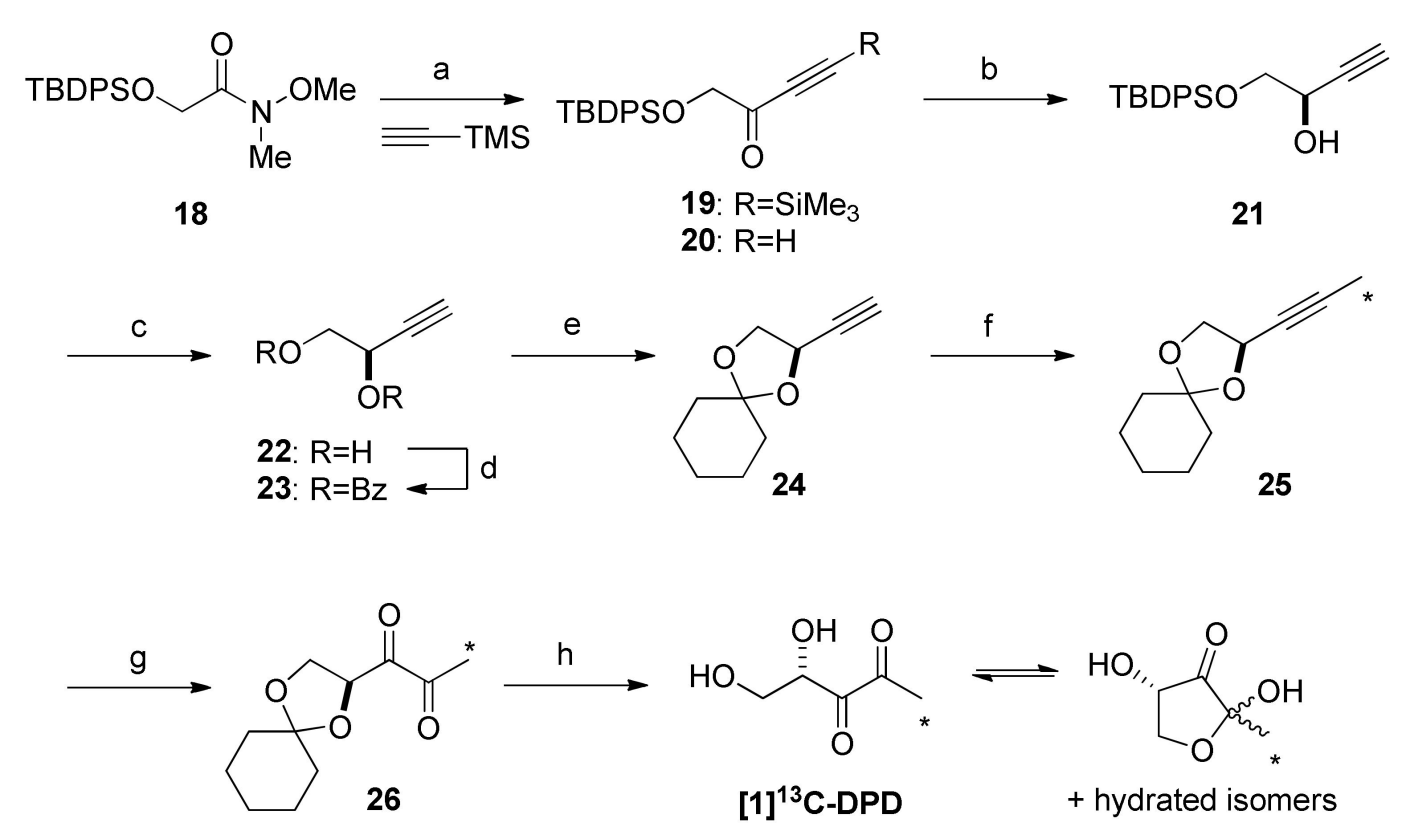
Synthesis of [1-^13^C]-DPD. Reagents and conditions: (**a**) LDA, THF, from −78 °C to 0 °C, 74%; (**b**) (*S*)-Alpine borane, THF, rt, 67%; (**c**) TBAF, THF, rt, 71%; (**d**) BzCl, (*i*-Pr)_2_NEt, DMAP, CH_2_Cl_2_, 96%; (**e**) 1,1-Dimethoxycyclohexane, H_2_SO_4_, DMF, 78%; (**f**) LDA, ^13^CH_3_I, THF, from −78 °C to rt, 94%; (**g**) NaIO_4_, RuO_2_, CCl_4_/MeCN, rt, 91%; (**h**) Dowex 50WX8, H_2_O, pH 3, rt.

It is also possible to prepare [1-^13^C]-labelled DPD by this method using [3-^13^C]- propyne or by alkylation of the terminal alkyne **24** (Semmelhack intermediate), prepared as described in Scheme 3, with ^13^CH_3_I [7].

Interestingly, reaction of the Weinreb amide **18** with lithium trimethylsilylacetylene afforded ketone **20** as the major product (62%) along with trimethylsilylacetylenic ketone **19** (12%) and 1,3-dione **27** (16%, Figure 1) [26]. Midland reduction of ketone **20** was chosen for this synthetic sequence, since terminal alkynes are strong inhibitors of Noyori’s reduction. Alcohol **21** was obtained with 66% yield and 86% *ee*. As diol **22** was not crystalline, the optical. purity of the product could not be enhanced by recrystallisation. Benzoylation of the diol did not produce a crystalline product.

The formation of the cyclohexylidene acetal **24**, alkylation of the terminal alkyne with ^13^CH_3_I afforded **25** and oxidation to the diketone **26** was successfully achieved as described previously [7]. Hydrolysis of the acetal with acidic Dowex resin afforded [1-^13^C]-labelled DPD (Scheme 3).

In conclusion, [4-^13^C]-AI-2 and [1-^13^C]-AI-2 were efficiently synthesised and the corresponding enantiomers obtained using the enantiomeric versions of the reduction reagents used. Noyori’s asymmetric transfer hydrogenation of α,β-acetylenic ketone **12** was employed for the first time in AI-2 synthesis, increasing the enantiomeric excess of the alcohol obtained and overcoming the difficult separation of pinene from (*S*)-Alpine borane previously used.

All reactions were reproducible and afforded stable intermediates in high yields. This is particularly relevant since ^13^C labelled compounds are expensive and, on the other hand, sufficient quantities of AI-2 and ^13^C labelled AI-2 are needed for biological studies.

AI-2 labelled at different carbon atoms is important to determine the metabolic fate of this molecule in vivo in bacteria where AI-2 metabolism remains to be discovered and to quantify potential incorporation of this molecule into central metabolism. Additionally, [^13^C]-AI-2 is also useful as a standard for the mass spectrometric quantification of AI-2 and derivatives in biological samples of in vivo animal studies.

## 3. Materials and Methods

^1^H-NMR spectra were obtained at 400 MHz in CDCl_3_ or D_2_O with chemical shift values (δ) in ppm downfield from tetramethylsilane in the case of CDCl_3_ and ^13^C-NMR spectra were obtained at 100.61 MHz in CDCl_3_ (see Supplementary Materials). Assignments are supported by 2D correlation NMR studies. Medium pressure preparative column chromatography: silica gel 60H. Analytical TLC: Aluminium-backed silica gel 60 F254. Specific rotations ([α]^20^_D_) were measured using an automatic polarimeter. Reagents and solvents were purified and dried according to the literature [27]. All reactions were carried out under an inert atmosphere (argon), except when the solvents were undried. The enantiomeric excesses were determined by HPLC on a Waters 600E/U6K instrument using a Daicel Chiralpack AD-H column (see Supplementary Materials).

*[1-^13^C]-2-bromo-N-methoxy-N-methylacetamide* (**8**). [1-^13^C]-2-bromoacetic acid (**7**, 510 mg, 3.64 mmol) was dissolved in dry toluene (18 mL), under argon atmosphere, and the solution was cooled to 0 °C. NHMe(OMe) (805 μL, 10.93 mmol) was added, and 10 min later a solution of PBr_3_ (173 μL, 1.82 mmol) in 4 mL of toluene was added dropwise. After stirring for 10 min at 0 °C, the reaction was warmed to room temperature, left stirring during 10 min and then warmed up to 60 °C. After the starting material was consumed (TLC, approximately 30 min), a saturated aqueous solution of NaHCO_3_ (35 mL) was added, and the aqueous phase was extracted with 3 portions of 18 mL of AcOEt. The combined organic layers were dried with MgSO_4_, filtered and concentrated under reduced pressure. The crude was purified by flash column chromatography (7:3 Hexane:AcOEt), and 586 mg of compound **8** was obtained as a colourless oil (Yield = 88%). ^1^H-NMR (400 MHz, CDCl_3_) δ (ppm): 4.02 (d, ^2^*J_H-C_* = 4.0 Hz, 2H, CH_2_), 3.80 (s, 3H, OMe), 3.24 (d, ^3^*J_H-C_* = 2.1 Hz, 3H, NMe). ^13^C-NMR (101 MHz, CDCl_3_) δ (ppm): 167.8 (C_1_), 61.8 (OCH_3_), 32.6 (NCH_3_), 25.3 (d, ^1^*J_C-C_* = 57.2 Hz, CH_2_). FT-IR (neat): 1618.6 (C=O). HRMS: calcd. for [C_3_^13^CH_8_BrNNaO_2_]^+^: 204.96642; found: 204.96647.

*[1-^13^C]-2-acetoxy-N-methoxy-N-methylacetamide* (**9**). To a solution of glacial AcOH (293 μL, 5.12 mmol) in dry 1,2-dichloroethane (2 mL), under argon atmosphere, Et_3_N (713 μL, 5.12 mmol) was added dropwise. Then, a solution of **8** (586 mg, 3.20 mmol) in dry 1,2-dichloroethane (2 mL) was added, and the reaction was heated at reflux temperature. Once the starting material was consumed (TLC, approximately 1 h), 50 mL of HCl 5% was added, and the aqueous phase was extracted with 3 portions of 55 mL of CH_2_Cl_2_. The combined organic layers were dried with MgSO_4_, filtered and concentrated under reduced pressure, rendering 446 mg of compound **19** that was used without any further purification (colourless oil, yield = 86%). ^1^H-NMR (400 MHz, CDCl_3_) δ (ppm): 4.83 (d, ^2^*J_C-H_* = 4.1 Hz, 2H, AcOCH_2_-), 3.74 (s, 3H, OMe), 3.20 (d, ^3^*J_C-H_* = 2.1 Hz, 3H, NMe), 2.18 (s, 3H, CH_3_C(O)O). ^13^C-NMR (101 MHz, CDCl_3_) δ (ppm): 170.8 (CH_3_C(O)O), 168.1 (C(O)N), 61.6 (OCH_3_), 61.1 (d, ^1^*J_C-C_* = 57.0 Hz, AcOCH_2_), 32.4 (NCH_3_), 20.7 (CH_3_C(O)O). FT-IR (neat): 1743.3 (AcO), 1639.1 (C(O)N). HRMS: calcd for [C_5_^13^CH_11_NNaO_4_]^+^: 185.06138; found: 185.06149.

*[1-^13^C]-2-hydroxy-N-methoxy-N-methylacetamide* (**10**). A solution of ester **9** (409 mg, 2.52 mmol) in dry MeOH (5.5 mL) was cooled to 0 °C under argon atmosphere, and MeONa (21 mg, 0.39 mmol) was added in small portions. The reaction was left to stir at 0 °C for 2 h, and then Dowex 50WX8 resin was added (until acidic pH). The resin was then filtered off, washed with methanol, and the solvent was removed under reduced pressure. 252 mg of compound **10** was obtained as a colourless oil, and it was used without any further purification (yield = 83%). ^1^H-NMR (400 MHz, CDCl_3_) δ (ppm): 4.31 (d, ^2^*J_H-C_* = 4.4 Hz, 2H, CH_2_OH), 3.71 (s, 3H, OMe), 3.24 (d, ^3^*J_C-H_* = 2.0 Hz, 3H, NMe). ^13^C-NMR (101 MHz, CDCl_3_) δ (ppm): 173.2 (C=O), 61.5 (OCH_3_), 59.8 (d, ^1^*J_C-C_* = 50.1 Hz, CH_2_OH), 32.50 (NMe). FT-IR (neat): 3434.4 (OH), 1614.8 (C(O)N). HRMS: calcd for [C_3_^13^CH_9_NNaO_3_]^+^: 143.05082; found: 143.05097.

*[1-^13^C]-2-(tert-Butyldiphenylsilyloxy)-N-methoxy-N-methylacetic acid* (**11**)**.** A solution of alcohol **10** (213 mg, 1.78 mmol) in dry CH_2_Cl_2_ (4 mL) was cooled to 0 °C under argon atmosphere, Et_3_N (494 μL, 3.55 mmol), TBDPSCl (554 μL, 2.13 mmol) and a catalytic amount of DMAP were added. The reaction was stirred at 0 °C during a 10 min period, and then it was left to stir overnight at room temperature. Then, a saturated aqueous solution of NH_4_Cl was added, and the aqueous phase was extracted with 3 portions of CH_2_Cl_2_. The combined organic layers were dried with MgSO_4_, filtered and concentrated under reduced pressure. The crude was purified by flash column chromatography (gradient: 5/95→10/90→20:80 AcOEt/Hexane), and 635 mg of compound **11** was obtained as a white solid (Yield = 100%). M.P. 54–55 °C (0.574 g). ^1^H-NMR (400 MHz, CDCl_3_) δ (ppm): 7.76–7.70 (m, 4H, H_ar-*m*_), 7.45–7.35 (m, 6H, H_ar-*o-p*_), 4.42 (d, ^2^*J_C-H_* = 4.1 Hz, 2H, CH_2_), 3.43 (s, 3H, OMe), 3.13 (d, ^3^*J_C-H_* = 2.1 Hz, 3H, NMe), 1.11 (s, 9H, *t*-Bu). ^13^C-NMR (101 MHz, CDCl_3_) δ (ppm): 171.8 (C=O), 135.7 (4 × C_arom_), 133.2 (2 × C_arom-quat_), 129.8 (2 × C_arom-p_), 127.7 (4 × C_arom_), 62.0 (d, ^1^*J_C-C_* = 56.1 Hz, CH_2_), 61.2 (OMe), 32.5 (NMe), 26.7 (*t*-Bu), 19.3 (*t*-Bu). FT-IR (neat): 1666.8 (C(O)N). HRMS: calcd. for [C_38_^13^C_2_H_54_N_2_NaO_6_Si_2_]^+^: 739.34797; found: 739.34843.

*[2-^13^C]-(tert-Butyldiphenylsilyloxy)pent-3-yne-2-one* (**12**)**.** To a solution of propyne (1.52 M in THF, 1.76 mL, 2.5 mmol) was slowly added *n*-BuLi (1.6 M in hexanes, 1.6 mL, 2.4 mmol) at −78 °C. After 30 min at −78 °C, the Weinreb amide **11** (0.748 g, 2.1 mmol) in THF was added. The reaction mixture was stirred at −78 °C for 20 min, and then at 0 °C for 1 h. Saturated NH_4_Cl solution (10 mL) was added and the mixture extracted with CH_2_Cl_2_ (3 × 10 mL) and the combined extracts dried (MgSO_4_), concentrated and purified by flash chromatography (30:70 EtOAc/hexane) to afford **12** as white crystals. M.P. 54–55 °C (0.638 g, 85%). ^1^H-NMR (400 MHz, CDCl_3_) δ (ppm): 7.69–7.66 (m, 4H), 7.44–7.37 (m, 6H), 4.30 (d, *J* = 4.1 Hz, 2H), 1.99 (d, *J* = 1.4 Hz, 3H), 1.10 (s, 9H). ^13^C-NMR (100 MHz, CDCl_3_) δ (ppm): 186.1, 135.6, 132.8, 129.9, 128.7, 93.3, 78.8, 70.5 (d, *J* = 49.7 Hz), 26.7, 19.3, 4.2.

*(2R)-2-[^13^C]-(tert-Butyldiphenylsilyloxy)pent-3-yne-2-ol* (**13**)**.** Ru(*p*-cymene)[(*S*,*S*)TsDPEN] catalyst was prepared in accordance with the procedure described in the literature [25]. To a degassed solution of ketone **12** (537 mg, 1.59 mmol) in dry *i*-PrOH (12 mL) was added Ru(*p*-cymene)[(*S*,*S*)TsDPEN] (72 mg, 0.0955 mmol, 0.06 eq.) in dry and degassed *i*-PrOH (3 mL) and the brown solution was left to stir at room temperature under argon atmosphere. After 2 h, the solvent was removed under reduced pressure and the crude was purified by flash chromatography (5:95 EtOAc/hexane) to afford **13** (513 mg, 95%, 94% *ee*). [α]^20^_D_ +7.48 (c 2.62, CH_2_Cl_2_). ^1^H-NMR (400 MHz, CDCl_3_) δ (ppm): 7.70–7.65 (m, 4H), 7.44–7.37 (m, 6H), 4.43 (dm, *J* = 148.6 Hz, 1H), 3.77 (dd, *J* = 3.8, 10.2 Hz, 1H), 3.70–3.66 (m, 1H), 1.81 (t, *J* = 1.6, 3H), 1.07 (s, 9H). ^13^C-NMR (100 MHz, CDCl_3_) δ (ppm): 135.6, 135.5, 133.0, 132.9, 129.9, 129.8, 127.8, 127.7, 82.0 (d, *J* = 12.7 Hz), 79.9, 67.9 (1H, *J* = 39.9 Hz), 79.9, 63.4, 26.8, 19.3, 3.5.

*(2R)-2-[^13^C]-Pent-3-yne-1,2-diol* (**14**)**.** To alcohol **13** (0.544 g, 1.6 mmol) in THF (3 mL) was added TBAF 1 M in THF (1.61 mL, 1.6 mmol) at r.t. The reaction mixture was stirred for 1h. Water (5 mL) was then added, and the aqueous phase was extracted with ethyl acetate (3 x 5 mL). The combined organic phases were concentrated, and the crude residue was purified by flash chromatography (70:30 EtOAc/hexane) to afford **14** as white crystals M.P. = 67–68 °C (0.114 g, 71%). [α]^20^_D_ −21.2 (c 0.99, CH_2_Cl_2_). ^1^H-NMR (400 MHz, CDCl_3_) δ (ppm): 4.42 (dm, *J* = 148.7 Hz, 1H), 3.71 (ddd, *J* = 0.64, 3.7, 11.3, 1H), 3.63 (ddd, *J* = 2.4, 6.64, 11.4, 1H), 1.86 (dd, *J* = 1.52, 1.88 Hz, 3H). ^13^C-NMR (100 MHz, CDCl_3_) δ (ppm): 81.7 (d, *J* = 12.4 Hz), 78.9, 66.7 (d, *J* = 38.5 Hz), 63.4, 3.5. HRMS: calcd. for C_4_H_8_O_2_Na^13^C 124.0450, found: 124.0453 (M + Na).

*(2R)-2-[^13^C]-Pent-1,2-benzoyloxy-3-yne* (**15**)**.** To the diol **14** (0.010 g, 0.1 mmol) in CH_2_Cl_2_ (1 mL) at 0 °C was added *N*-ethyldiisopropylamine (0.070 mL, 0.4 mmol), benzoyl chloride (0.035 mL, 0.3 mmol) and a catalytic amount of DMAP. The mixture was stirred at room temperature until total consumption of the starting material. A saturated solution of NaHCO_3_ was added and the resulting mixture was extracted with CH_2_Cl_2_, concentrated under vacuum and purified by flash chromatography (70:30 EtOAc/hexane) to give **15** as white crystals (0.028 g, 91% yield) M.P. = 70 °C. [α]^20^_D_ −46.3 (c 1.35, CH_2_Cl_2_). ^1^H-NMR (400 MHz, CDCl_3_) δ (ppm): 8.07 (d, *J* = 7.2 Hz, 2H), 8.01 (d, *J* = 7.1 Hz, 2H), 7.58–7.52 (m, 2H), 7.45–7.39 (m, 4H), 5.96 (dm, *J* = 154 Hz, 1H), 4.65–4.63 (m, 2H), 1.87 (t, *J* = 1.44 Hz, 3H). ^13^C-NMR (100 MHz, CDCl_3_) δ (ppm): 166.04 (d, *J* = 2 Hz), 165.5 (d, *J* = 2 Hz), 133.2, 133.1, 129.8, 129.7, 129.63, 129.60, 128.42, 84.0 (d, *J* = 13 Hz), 79.5, 73.5, 72.7, 65.4 (d, *J* = 42.2 Hz), 63.0, 3.7. HRMS: calcd. for C_18_H_16_O_4_Na^13^C 332.0974, found: 332.0972 (M + Na).

*(2R)-2-[^13^C]-1,2-Cyclohexylidenedioxypent-3-yne* (**16**)**.** To diol **14** (0.059 mg, 0.6 mmol) in DMF (2.0 mL) at room temperature, was added 1,1-dimethoxycyclohexanone (0.269 mL, 1.8 mmol) and H_2_SO_4_ (1 drop), and the reaction mixture was stirred overnight. The reaction was quenched with NaHCO_3_ sat. solution (2 mL) (pH ≥ 8). The mixture was extracted with Et_2_O (3 × 2 mL), concentrated and purified by flash chromatography (5:95 EtOAc/hexane) to afford **16** (94 mg, 89%) as a colourless oil. [α]^20^_D_ −37.6 (c 1.31, CHCl_3_). ^1^H-NMR (400 MHz, CDCl_3_) δ (ppm): 4.67 (dm, *J* = 153.6 Hz, 1H), 4.10 (dd, *J* = 1.68, 7.8 Hz, 1H), 3.81 (ddd, *J* = 2.4, 7.1, 14.8 Hz, 1H), 1.85 (d, *J* = 1.8 Hz, 3H), 1.74–1.39 (m, 10H). ^13^C-NMR (100 MHz, CDCl_3_) δ (ppm): 110.4, 82.1 (d, *J* = 13.9 Hz), 75.9, 69.6 (d, *J* = 32.9 Hz), 65.5, 35.7, 35.4, 25.0, 23.8, 23.7, 22.8, 3.6. HRMS: calcd. for C_10_H_15_O_2_^13^C 180.1100, found: 180.1101 (M-H).

*(4S)-4-[^13^C]-4,5-Cyclohexylidenedioxy-2,3-pentadione* (**17**)**.** To compound **16** (106 mg, 0.6 mmol) in CCl_4_ (3.2 mL) and MeCN (3.2 mL) was added NaIO_4_ (286 mg, 1.3 mmol) in H_2_O (4.8 mL) and RuO_2_.H_2_O (2.0 mg, 0.015 mmol), the reaction mixture was vigorously stirred until all starting material has been consumed (TLC). The mixture was extracted with ethyl acetate (3 × 10 mL), filtred by silica gel and concentrated to afford a yellow oil **17** (103 mg, 82%). [α]^20^_D_ −11.5 (c 0.8, CHCl_3_). ^1^H-NMR (400 MHz, CDCl_3_) δ (ppm): 5.13 (ddd, *J* = 5.3, 7.9, 153.5 Hz, 1H), 4.35 (t, *J* = 8.4 Hz, 1H), 4.0 (dd, *J* = 5.3, 8.9 Hz, 1H), 2.39 (s, 3H), 1.74–1.34 (m, 10H). ^13^C-NMR (100 MHz, CDCl_3_) δ (ppm): 198.1, 194.7, 111.9, 76.5, 65.5 (d, *J* = 34.6 Hz), 35.4, 34.7, 24.9, 24.5, 23.8, 23.7.

*(4S)-4-[^13^C]-DPD* (**1**)**.** The experimental procedure described in ref. 8 was followed. ^1^H NMR (500 MHz, CDCl_3_) δ (ppm): 4.05 (ddd, *J* = 149.6, 7.0, 5.8 Hz, H4, cyclic form), 3.87–3.82 (m), 3.70 (dm, *J* = 145 Hz, H4, cyclic form), 3.65 (dm, *J* = 145 Hz, H4, linear form), 3.52–3.47 (m), 3.24 (ddd, *J* = 10.95, 5.4, 5.4 Hz), 2.04 (CH_3_, linear form), 1.11 (CH_3_, cyclic form), 1.08 (CH_3_, cyclic form). ^13^C-NMR (100 MHz, CDCl_3_) δ (ppm): 74.0 (C4, cyclic form), 73.8 (C4, linear form), 73.2 (C4, cyclic form).

*1-(tert-Butyldiphenylsilyloxy)but-3-yne-2-one* (**19**)**.** To a solution of *N*-diisopropylamine (1.25 mL, 8.09 mmol) in THF (13.6 mL), was added *n*-Buli (1.6M in hexane, 4.23 mL, 6.76 mmol) dropwise at 0 °C, and stirred at 0 °C over 10 min. At −78 °C, it was added trimethylsilylacetylene (0.99 mL, 7.1 mmol), after 30 min Weinreb amide **18** was added (2.12 g, 5.93 mmol), previously dissolved in THF (17.3 mL). The mixture was stirred at −78 °C and warmed to room temperature. Water was added (20 mL), the resulting mixture was extracted with CH_2_Cl_2_ (3 × 15 mL), dried with anhydrous MgSO_4_, concentrated under vacuum and purified by flash chromatography (30:70 AcOEt:hexane) to afford colourless oil **20** (0.850 g, 62%), **19** (0.107 g, 12% yield) and **27** (0.330 g, 16% yield). Data for **19**: ^1^H-NMR (400 MHz, CDCl_3_) δ (ppm): 7.70–7.67 (m, 4H), 7.44–7.37 (m, 6H), 4.29 (s, 2H), 1.10 (s, 9H), 0.22 (s, 9H). Data for **20**: ^1^H-NMR (400 MHz, CDCl_3_) δ (ppm): 7.68–7.65 (m, 4H), 7.45–7.38 (m, 6H), 4.34 (s, 2H), 3.26 (s, 1H), 1.10 (s, 9H). ^13^C-NMR (100 MHz, CDCl_3_) δ (ppm): 185.3, 135.5, 132.5, 130.0, 127.9, 81.2, 79.6, 70.6, 26.6, 19.3. FT-IR (film): 2094 (C≡C), 1706 (C=O). Data for **27**: ^1^H-NMR (400 MHz, CDCl_3_) δ (ppm): 7.84 (d, *J* = 12.9 Hz, 1H), 7.69–7.67 (m, 4H), 7.43–7.34 (6H, m), 5.92 (d, *J* = 12.9 Hz, 1H), 4.17 (s, 2H), 1.11 (s, 9H). ^13^C-NMR (100 MHz, CDCl_3_) δ (ppm): 196.2, 148.3, 135.5, 133.3, 129.7, 127.7, 90.7, 68.9, 26.8, 19.3.

*(2R)-1-(tert-butyldiphenylsilyloxy)but-3-yn-2-ol* (**21**)**.** Acetylenic ketone **19** (1 g, 3.1 mmol) was dissolved in THF (10 mL) at room temperature. (*S*)-Alpine Borane (0.5 M in THF, 9.3 mL, 4.65 mmol) was added, the mixture was stirred for 36 h. NH_4_Cl saturated solution (15 mL) was added, the resulting mixture was extracted one time with ethyl ether (10 mL) and then with dichloromethane (2 × 10 mL), dried with anhydrous MgSO_4_, concentrated under vacuum and purified by flash chromatography (30:70 AcOEt:hexane and 1:12:12 EtOAc:hexanes:CH_2_Cl_2_) to give **21** (0.675 g, 67% yield). Usually, no purification was done as it was very difficult to isolate the product. ^1^H-NMR (400 MHz, CDCl_3_) δ (ppm): 7.69–7.65 (m, 4H), 7.44–7.37 (m, 6H), 4.47–4.44 (m, 1H), 3.81 (dd, *J* = 10.2, 3.9 Hz, 1H), 3.74 (dd, *J* = 10.2, 6.5 Hz, 1H), 2.41 (d, *J* = 2.1 Hz, 1H), 1.08 (s, 9H). ^13^C-NMR (100 MHz, CDCl_3_) δ (ppm): 135.6, 135.5, 132.8, 132.7, 129.9, 127.87, 127.85, 81.8, 73.6, 67.4, 63.0, 26.8, 19.3. FT-IR (film): 3427 (O-H). To quantify the enantioselectivity of the reaction, a small sample of alcohol **21** was treated with (*R*)-MTPA-Cl (3 eq) and DMAP (3 eq) in CH_2_Cl_2_ at room temperature. Integration of ^1^H-NMR (400 MHz, CDCl_3_) peaks at δ (ppm): 2.50 (d, *J* = 2.2 Hz, minor) and 2.43 (d, *J* = 2.2 Hz, major) ppm indicated a dr of 93:7 corresponding to 86% *ee*.

*(2R)-But-3-yne-1,2-diol* (**22**)**.** To the alcohol **21** (0.5 g, 1.54 mmol) in THF (5 mL) was added TBAF (1M in THF, 1.85 mL, 1.85 mmol) at room temperature. The reaction mixture was stirred until all starting material has been consumed (TLC) and then quenched with H_2_O (5 mL). The resulting mixture was extracted with ethyl acetate (3 × 5 mL), concentrated under vacuum and purified by flash chromatography (70:30 EtOAc:hexane) to give **22** as a colourless liquid (0.094 g, 71% yield). [α]^20^_D_ = −27.4 (c 1.92, CH_2_Cl_2_). ^1^H-NMR (400 MHz, CDCl_3_) δ (ppm): 4.49–4.46 (m, 1H), 3.77 (dd, *J* = 11.4, 3.7 Hz, 1H), 3.70 (dd, *J* = 11.4, 6.5 Hz, 1H), 2.49 (d, *J* = 2.1 Hz, 1H). ^13^C-NMR (100 MHz, CDCl_3_) δ (ppm): 81.8, 74.1, 66.4, 63.0. FT-IR (film): 3291 (O-H), 2117 (C≡C).

*(2R)-1,2-Cyclohexylidenedioxybut-3-yne* (**24**)**.** To the diol **22** (0.094 g, 1.09 mmol) in DMF (2 mL) at room temperature was added 1,1-dimethoxycyclohexane (0.665 mL, 4.37 mmol) and one drop of conc. H_2_SO_4_ and stirred overnight. NaHCO_3_ saturated solution was added (pH ≥ 8) followed by H_2_O (5 mL). The mixture was extracted with ethyl ether (3 × 8 mL), concentrated under vacuum and purified by flash chromatography (5:95 EtOAc:hexane) to afford colourless oil **24** (0.141 g, 78% yield). ^1^H-NMR (400 MHz, CDCl_3_) δ (ppm): 4.71 (ddd, *J* = 6.3, 6.3, 2.0 Hz, 1H), 4.16 (dd, *J* = 8.4, 6.3 Hz, 1H), 3.94 (dd, *J* = 8.0, 6.2 Hz, 1H), 2.48 (d, *J* = 2.2 Hz, 1H), 1.77–1.59 (m, 8H), 1.42–1.40 (m, 2H). ^13^C-NMR (100MHz, CDCl_3_) δ (ppm): 111.2, 81.6, 73.7, 69.5, 64.9, 35.6, 35.4, 27.0, 25.0, 23.8. FT-IR (film): 2119 (C≡C).

*(2R)-5-[^13^C]-1,2-Cyclohexylidenedioxypent-3-yne* (**25**)**.** To a solution of *N,N-*diisopropylamine (0.298 mL, 2.1 mmol) in THF (3.23 mL), was added *n*-Buli (1.6 M in hexane, 1.06 mL, 1.7 mmol) at 0 °C and stirred for 10 min. At −78 °C, **24** (0.141 g, 0.848 mmol), previously dissolved in THF (1.15 mL) was added. After 30 min, ^13^CH_3_I (0.133 mL, 2.1 mmol) was added. The mixture was stirred at −78 °C and allowed to warm to room temperature. NH_4_Cl saturated solution was added (5 mL), the resulting mixture was extracted with CH_2_Cl_2_ (3 × 5 mL), dried with anhydrous MgSO_4_, concentrated under vacuum and purified by flash chromatography (50:50 AcOEt:hexane) to afford colourless oil **25** (0.150 g, 94% yield). ^1^H-NMR (400 MHz, CDCl_3_) δ (ppm): 4.69–4.66 (m, 1H), 4.11 (dd, *J* = 7.9, 6.2 Hz, 1H), 3.82 (dd, *J* = 7.8, 7.0 Hz, 1H), 1.85 (dd, *J* = 131.5, 2.0 Hz, 3H), 1.67–1.58 (m, 8H), 1.41–1.38 (m, 2H).

*(4S)-1-[^13^C]-4,5-cyclohexylidenedioxy-2,3-pentadione* (**26**)**.** To the alkyne **25** (0.083 g, 0.462 mmol) dissolved in CCl_4_ (2.5 mL) and MeCN (2.5 mL) was added a solution of NaIO_4_ (0.224 g, 1.05 mmol) in H_2_O (3.8 mL) and RuO_2_·H_2_O (0.0015 g, 0.0115 mmol) and the reaction mixture was stirred vigorously for 15 min. The mixture was extracted with CH_2_Cl_2_, filtered by a short pad of silica for medium pressure chromatography and concentrated under vacuum to give the bright yellow oil **26** (0.089 g, 91% yield) with NMR data identical to that described in the literature[5].^1^H-NMR (400 MHz, CDCl_3_) δ (ppm): 5.13 (dd, *J* = 7.9, 5.3 Hz, 1H), 4.35 (dd, *J* = 8.9, 7.9 Hz, 1H), 4.00 (dd, *J* = 8.9, 5.3 Hz, 1H), 2.38 (d, *J* = 129.3 Hz, 3H), 1.77–1.38 (m, 10H).

*(4S)-1-[^13^C]-DPD* (**1**)**.** The experimental procedure described in ref. [8] was followed. The characterisation data are in accordance with those described in ref. [5]

## Supplementary Materials

The following are available online: Figures S1 and S2: HPLC chromatogram of racemic **15** and HPLC chromatogram of optically active **15**. Figures S3–S39: NMR spectra.

## Sample Availability

Samples of the compounds are available for purchase from the authors.
