# Supplementary material for: An Efficient Synthesis of Optically Active [4-13C] Labelled Quorum Sensing Signal Autoinducer-2
Source: Molecules. 2021 Jan 12;26(2):369. doi: 10.3390/molecules26020369 (PMC7828210; doi:10.3390/molecules26020369)
Supplement: Supplementary file 1 [file molecules-26-00369-s001.pdf]

Supporting information

An Efficient Synthesis of Optically Active [4]^{13}C Labelled Quorum Sensing Signal Autoinducer-2

Osvaldo S. Ascenso,[a] Gonzalo Carrau,[a,b] Karina B. Xavier,[c] M. Rita Ventura,[a*] Christopher D. Maycock[a,d*]

[a]Instituto de Tecnologia Química e Biológica António Xavier, Universidade Nova de Lisboa, Apartado 127, 2780-901 Oeiras, Portugal.

[b]Facultad de Química, Universidad de la República, Montevideo, Uruguay.
[c]Instituto Gulbenkian de Ciência, 2781-901 Oeiras, Portugal.

[d]Faculdade de Ciências da Universidade de Lisboa, Departamento de Química e Bioquímica, 1749-016 Lisboa, Portugal.

1). HPLC Chromatograms.

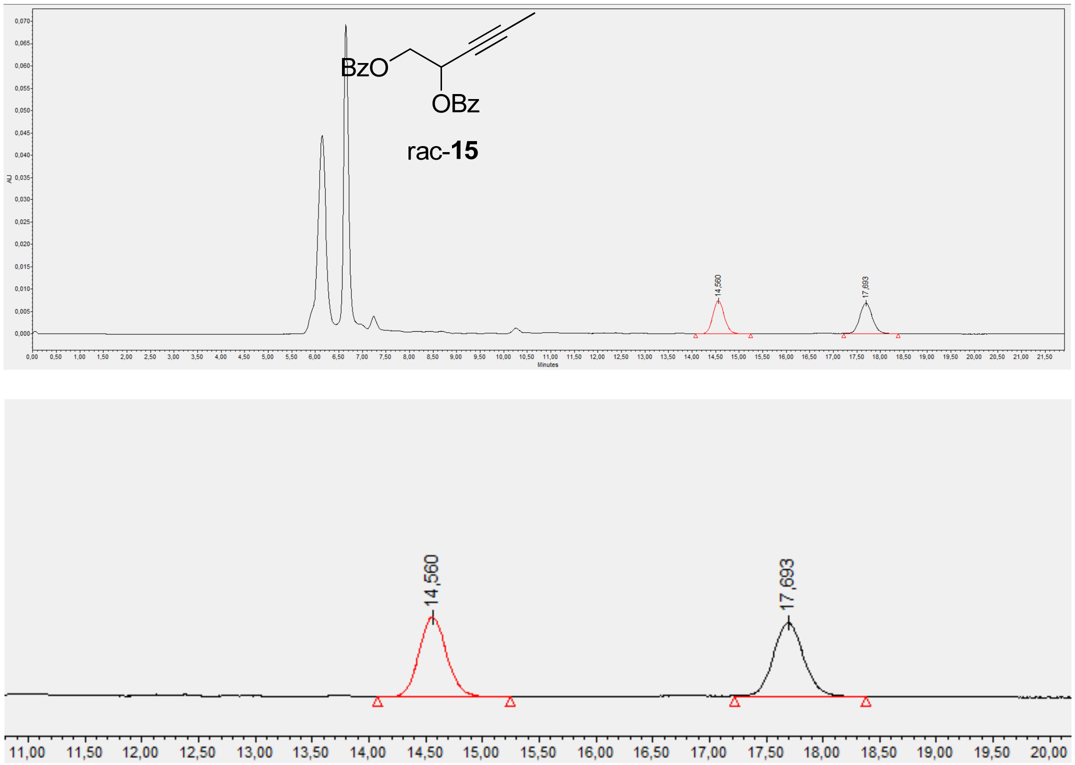

Figure S1. – Chromatogram of racemic **15.**

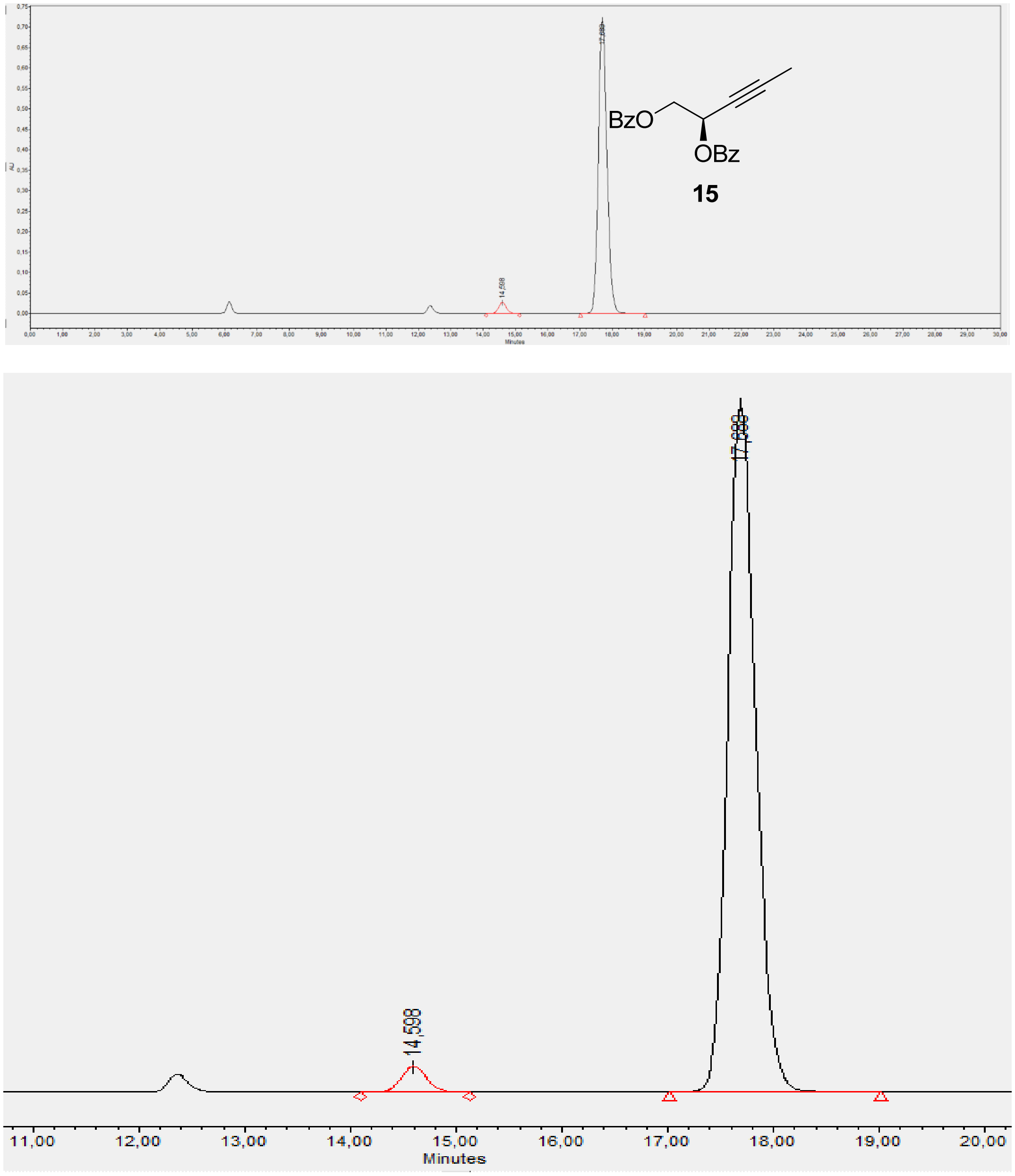

Figure S2. – Chromatogram of optically active **15** (94% *ee*).

2). NMR Spectra

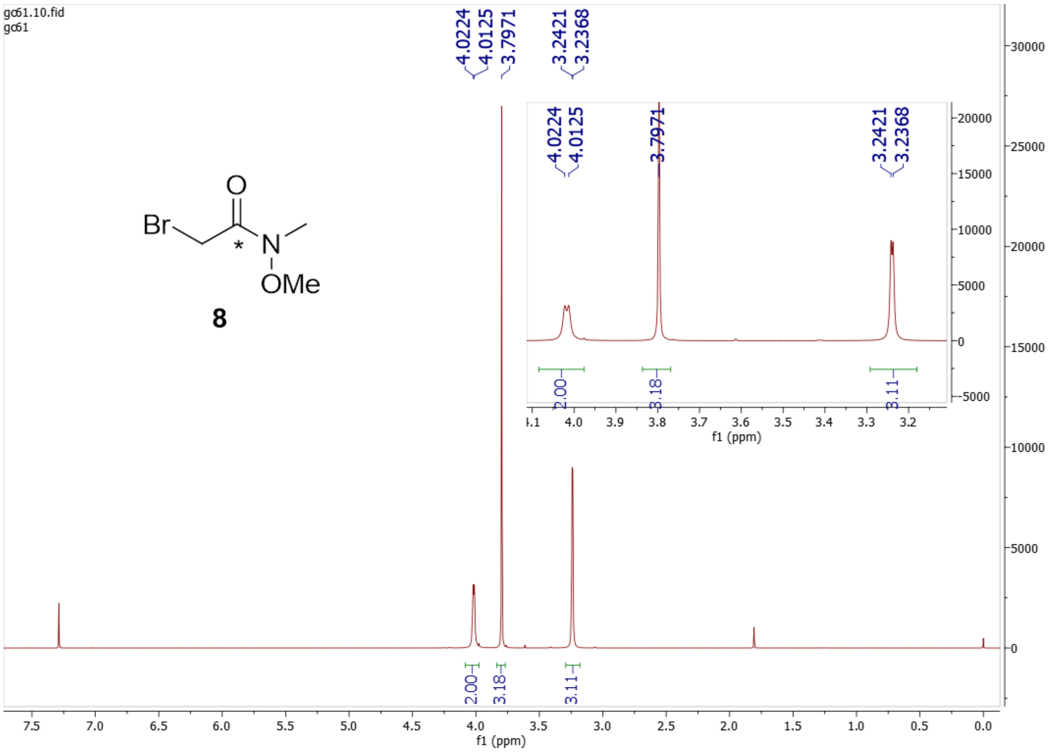

Figure S3. – ^{1}H-NMR spectrum of compound **8** in CDCl3.

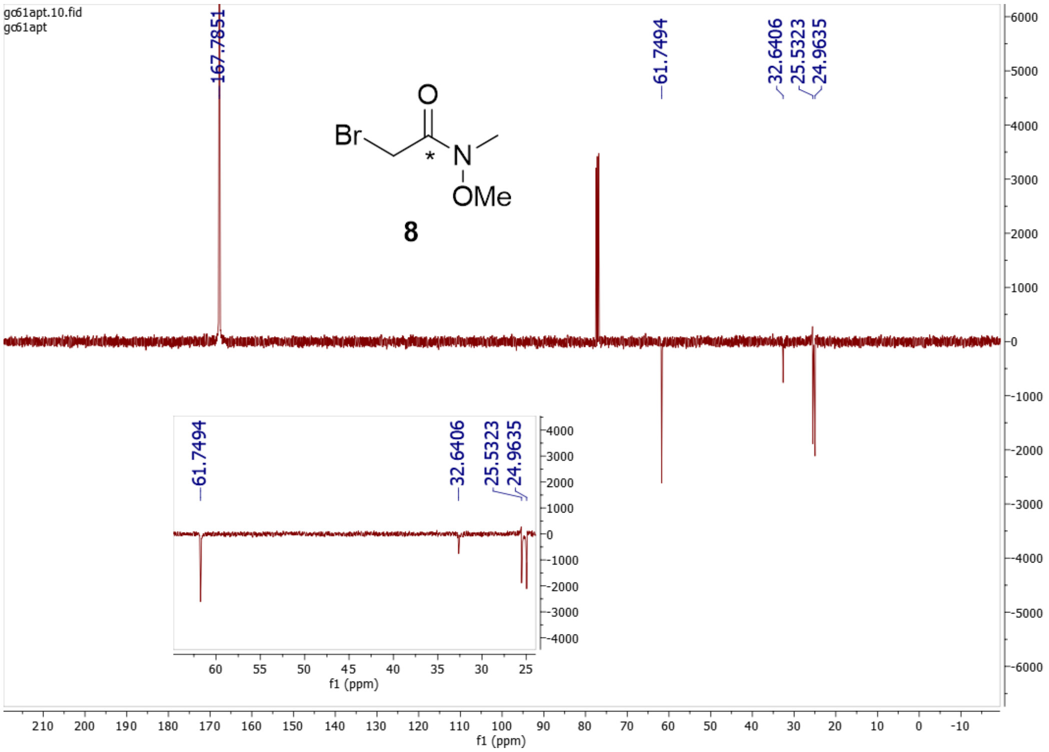

Figure S4. – APT spectrum of compound **8** in CDCl3.

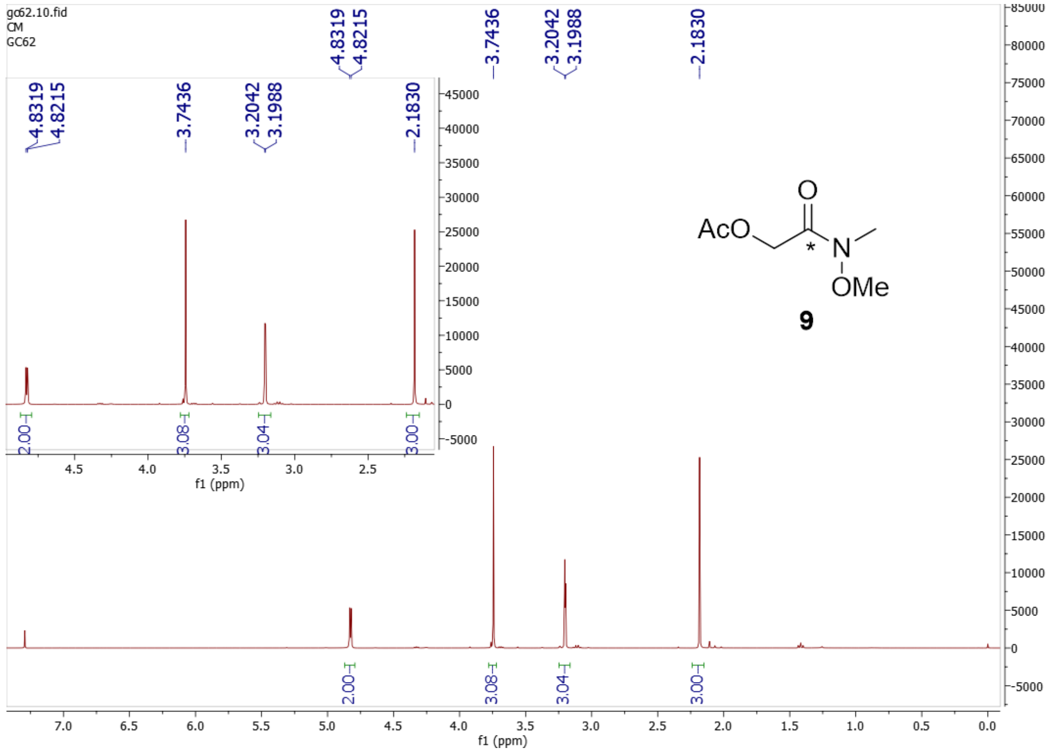

Figure S5. – ^{1}H-NMR spectrum of compound **9** in $CDCl_3$.

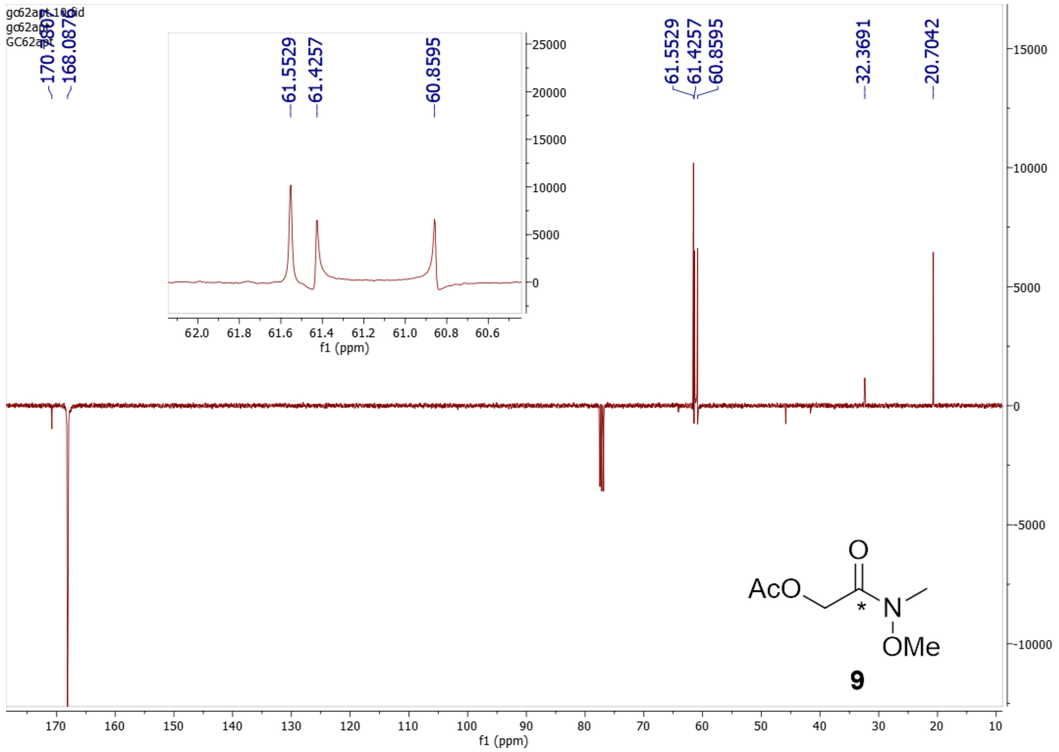

Figure S6. – APT spectrum of compound **9** in $CDCl_3$.

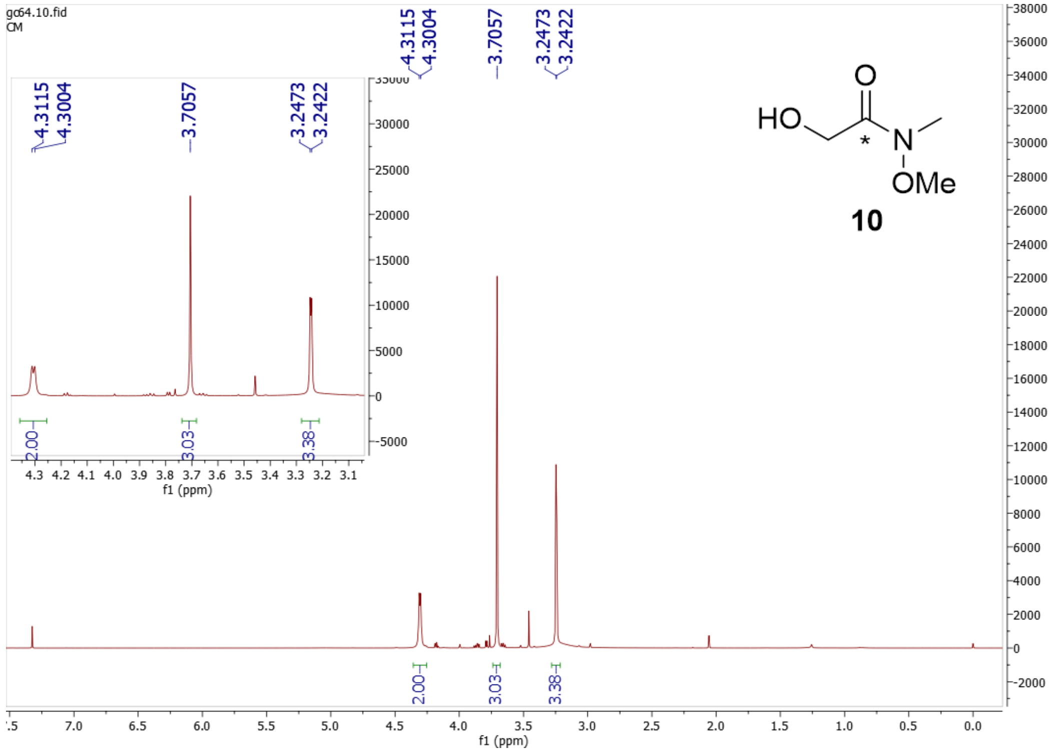

Figure S7. – ^{1}H-NMR spectrum of compound **10** in $CDCl_3$.

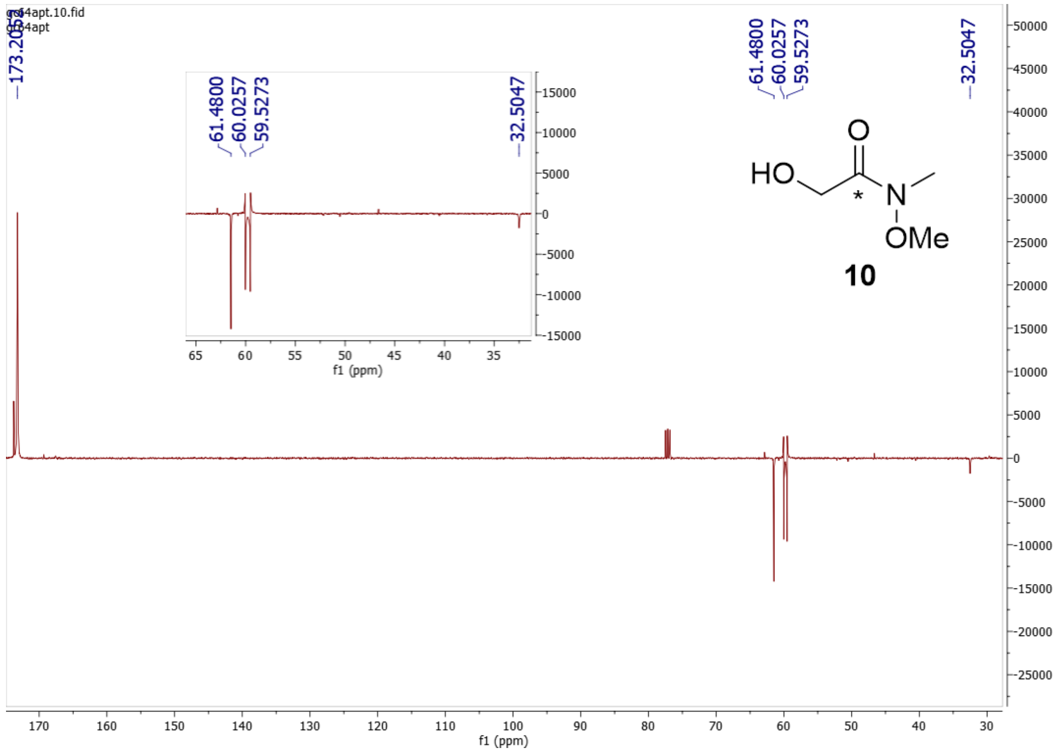

Figure S8. – APT spectrum of compound **10** in $CDCl_3$.

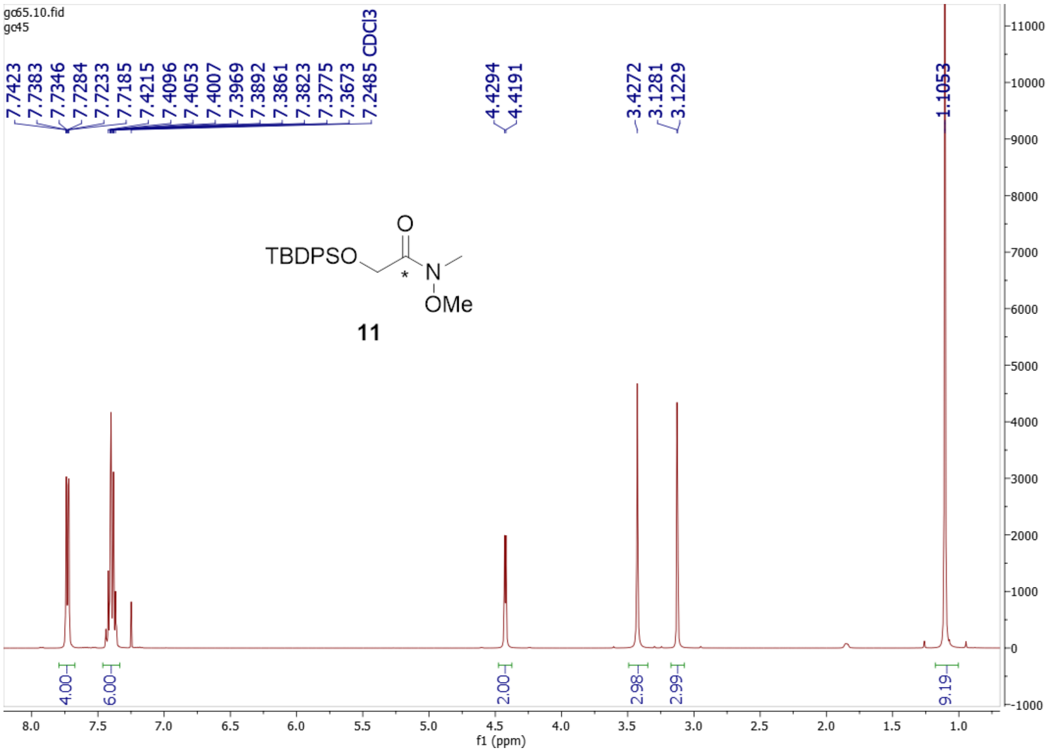

Figure S9. – ^{1}H-NMR spectrum of compound **11** in $CDCl_3$.

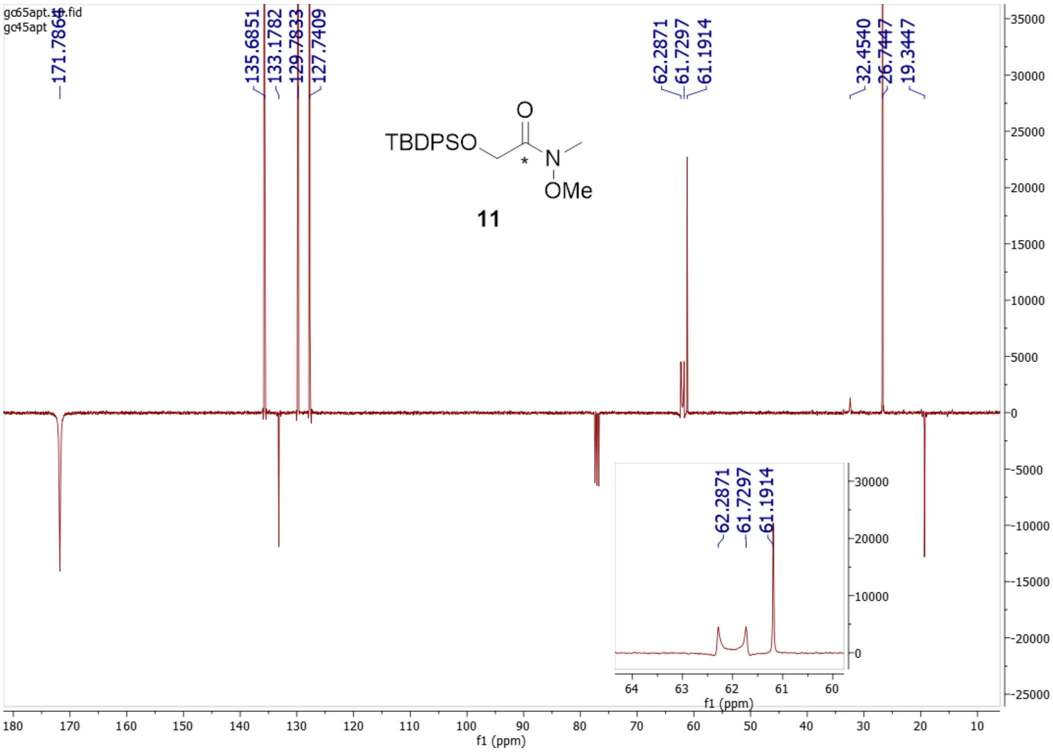

Figure S10. – APT spectrum of compound **11** in $CDCl_3$.

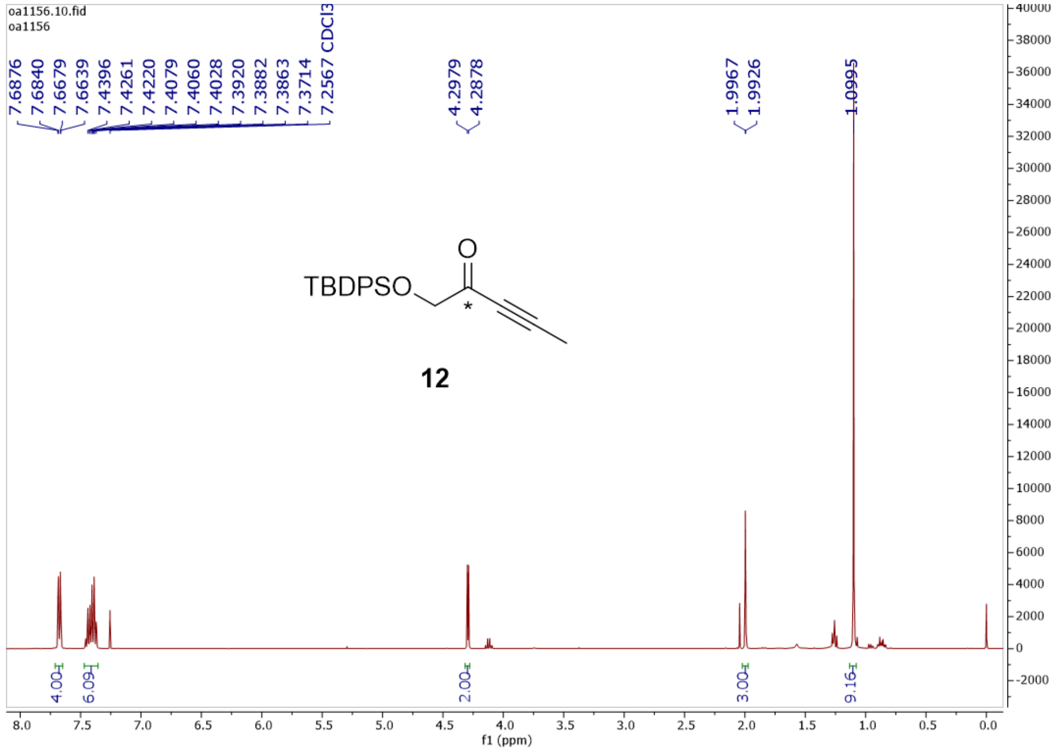

Figure S11. – ^{1}H-NMR spectrum of compound **12** in $CDCl_3$.

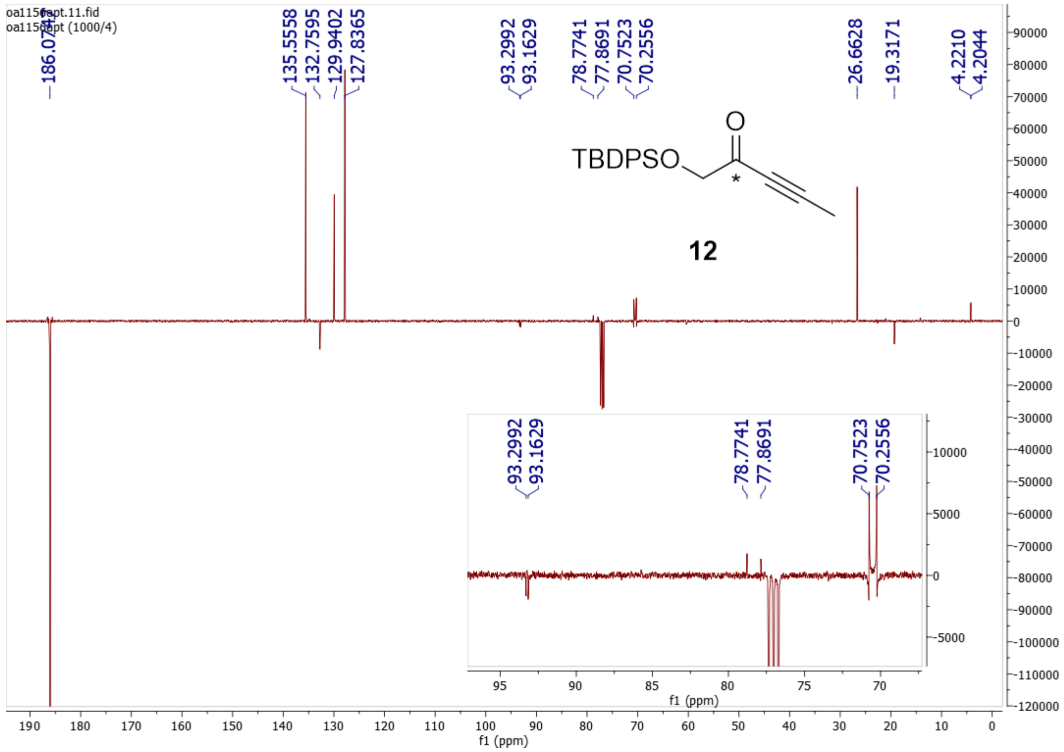

Figure S12. – APT spectrum of compound **12** in $CDCl_3$.

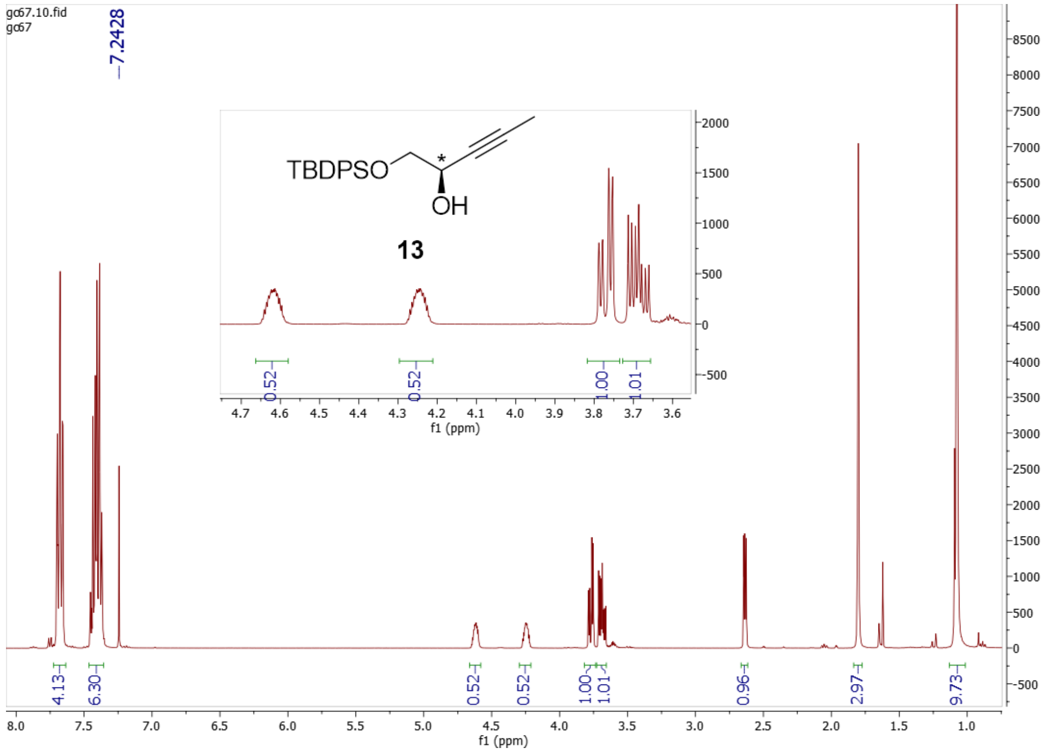

Figure S13. – ^{1}H-NMR spectrum of compound **13** in $CDCl_3$.

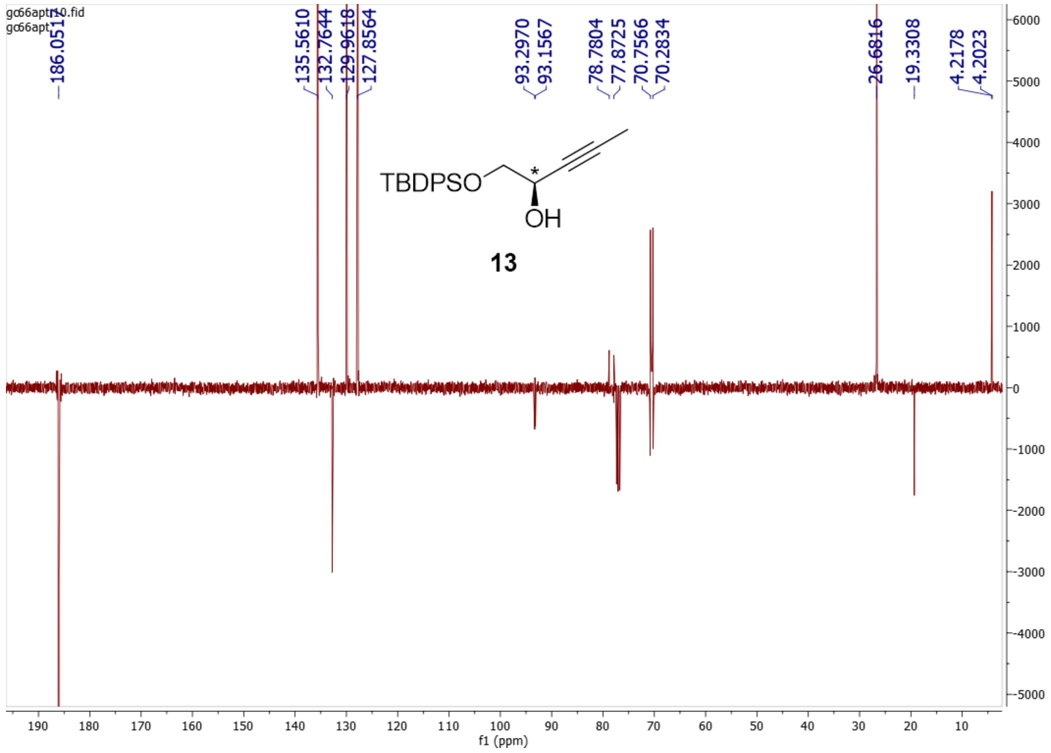

Figure S14. – ^{13}C-NMR spectrum of compound **13** in $CDCl_3$.

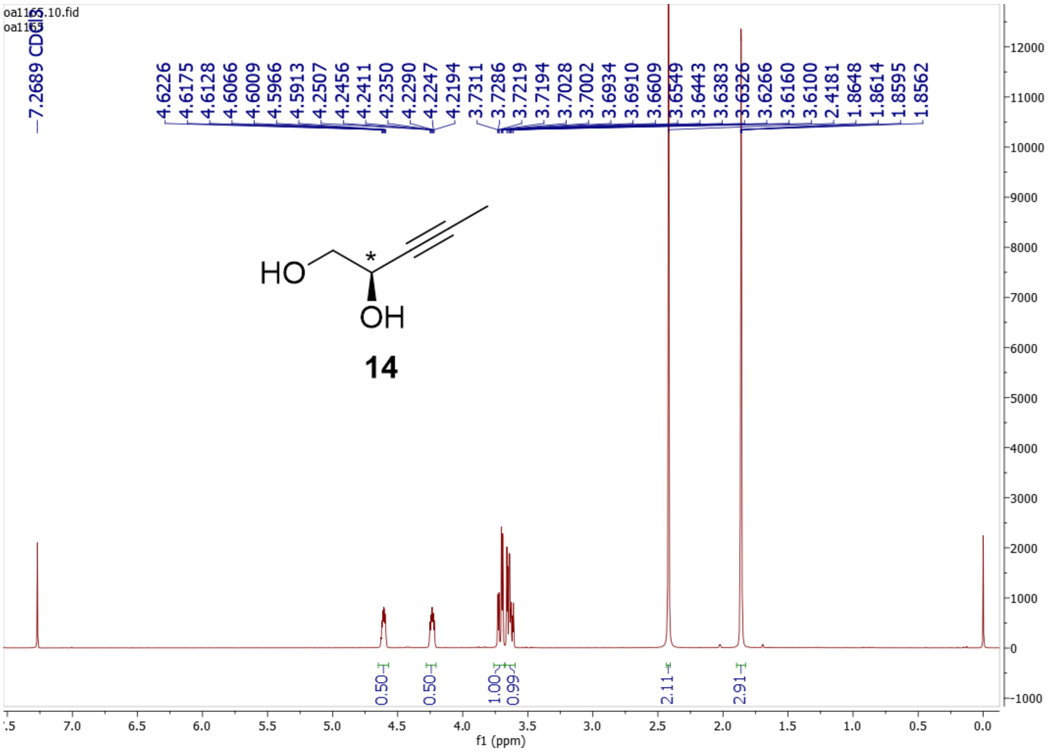

Figure S15. – ^{1}H-NMR spectrum of compound **14** in $CDCl_3$.

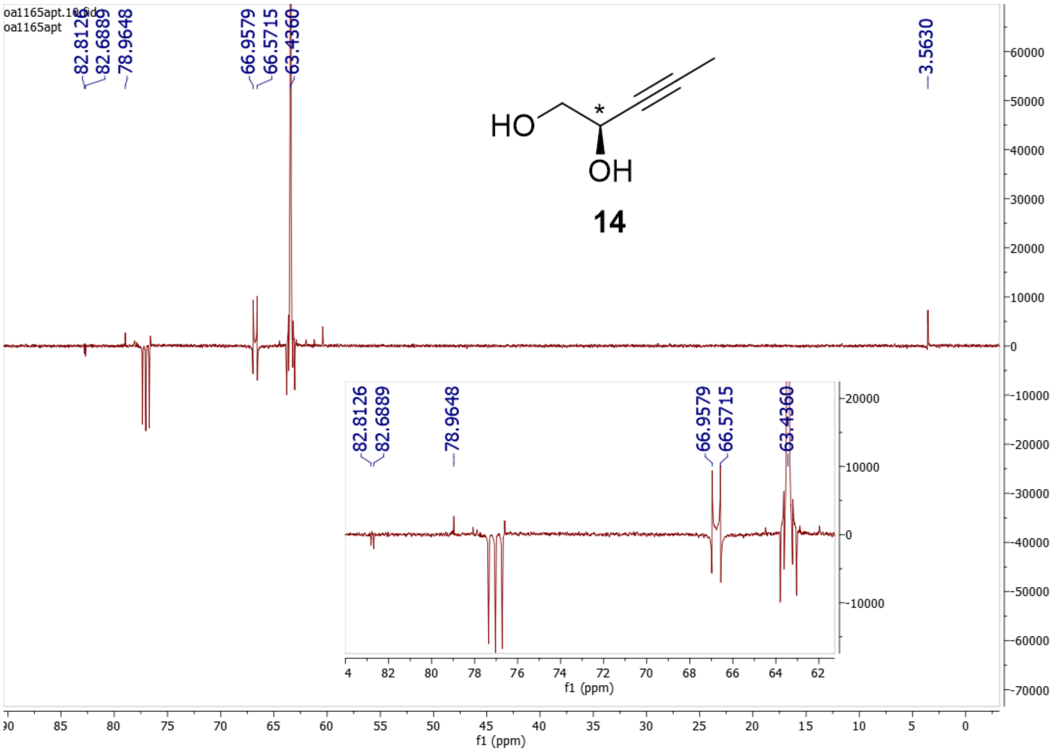

Figure S16. – APT spectrum of compound **14** in $CDCl_3$.

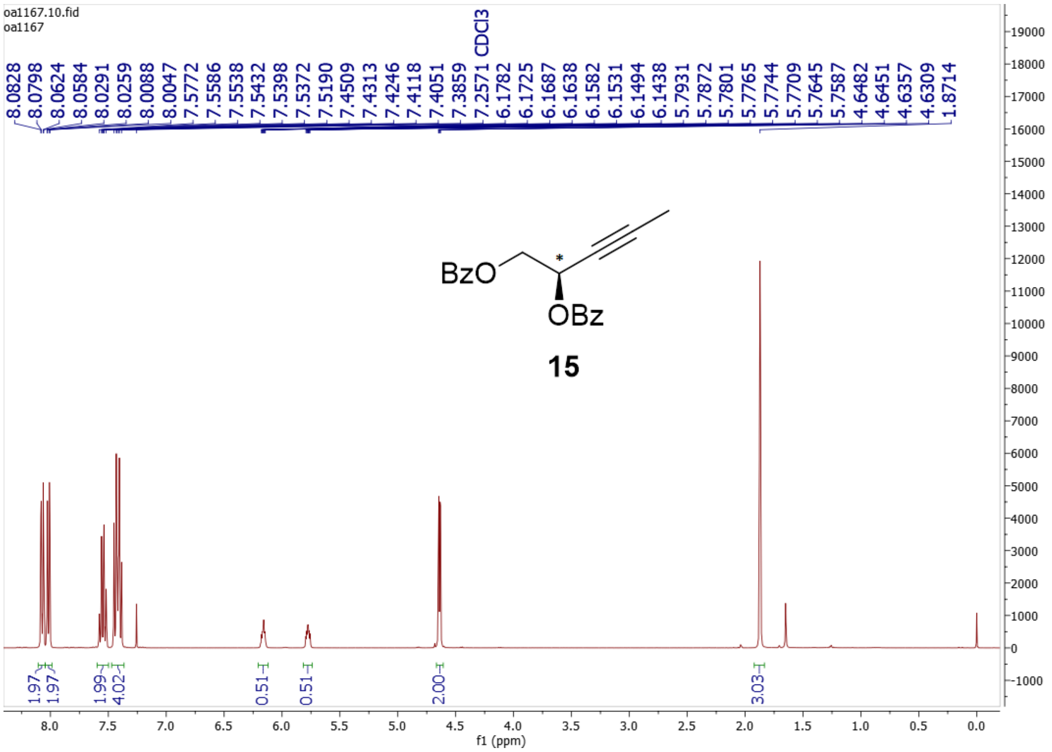

Figure S17. – ^{1}H-NMR spectrum of compound **15** in $CDCl_3$.

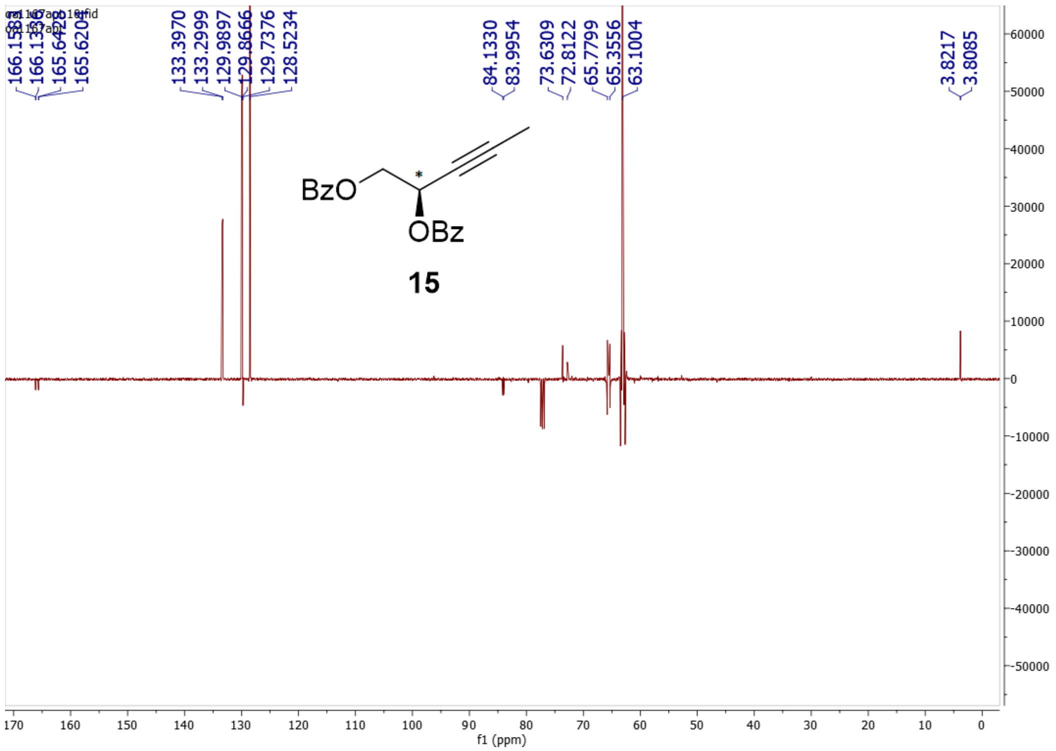

Figure S18. – APT spectrum of compound **15** in $CDCl_3$.

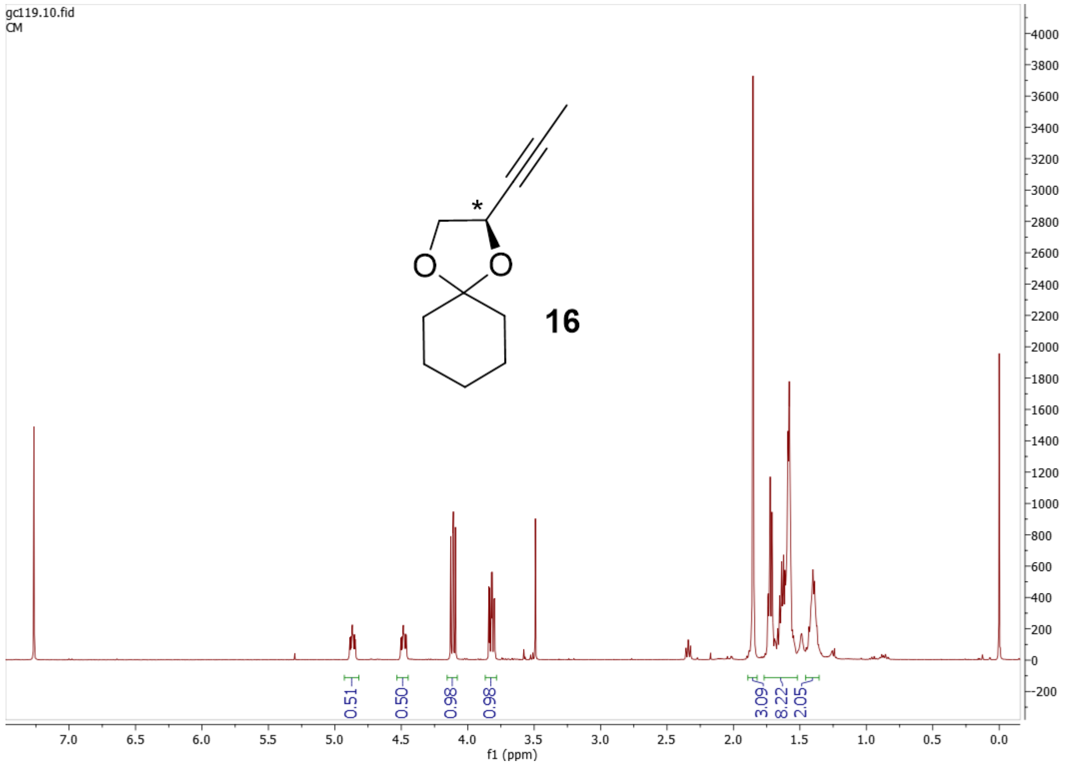

Figure S19. – ^{1}H-NMR spectrum of compound **16** in $CDCl_3$.

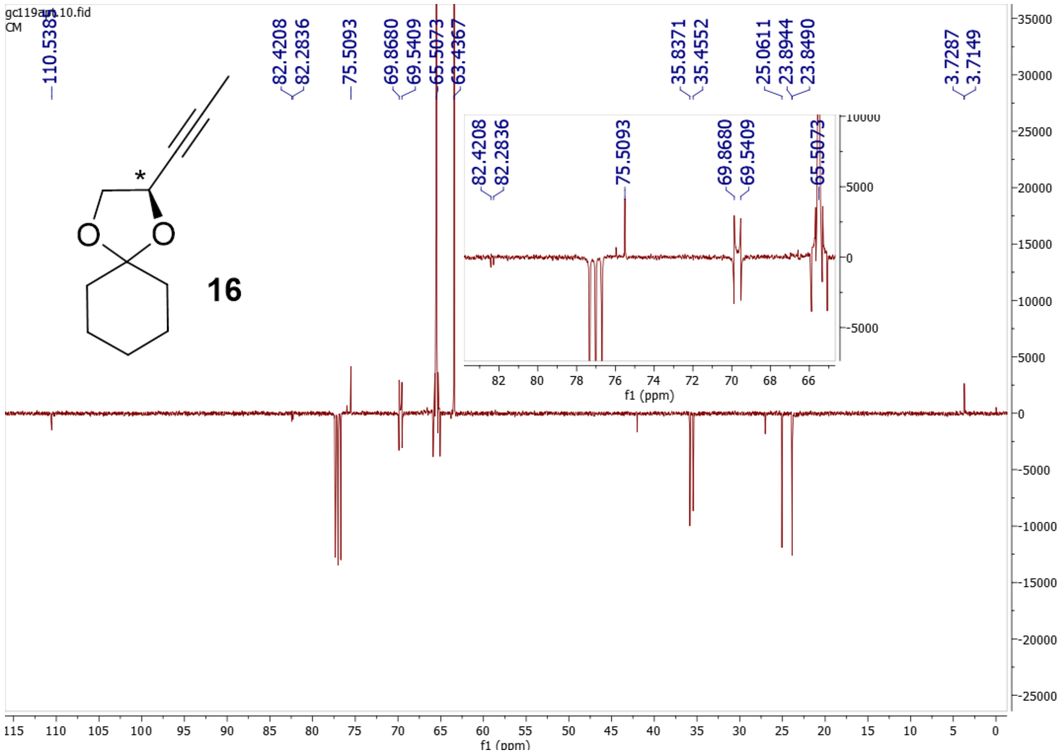

Figure S20. – APT spectrum of compound **16** in $CDCl_3$.

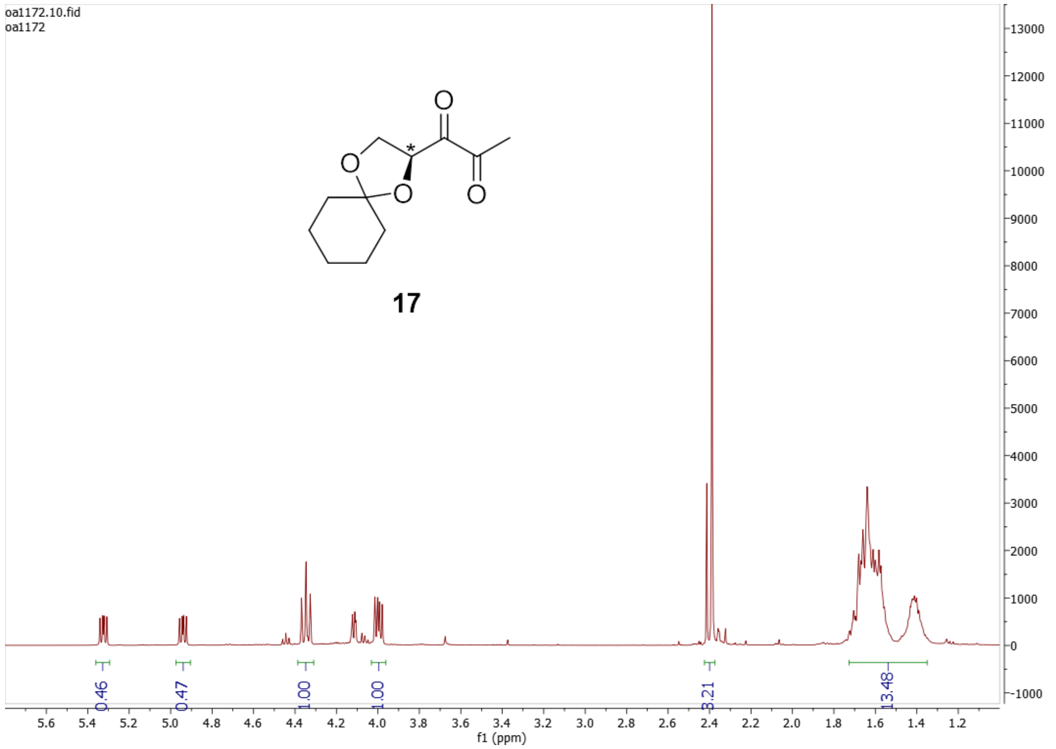

Figure S21. – ^{1}H-NMR spectrum of compound **17** in $CDCl_3$.

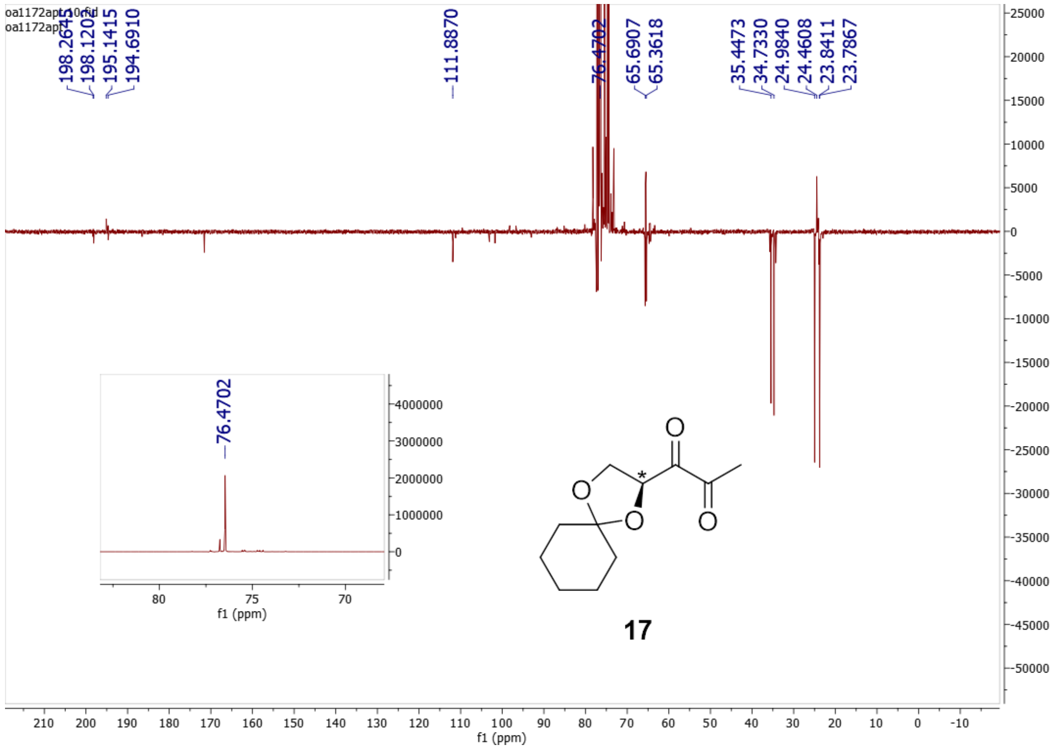

Figure S22. – APT spectrum of compound **17** in $CDCl_3$.

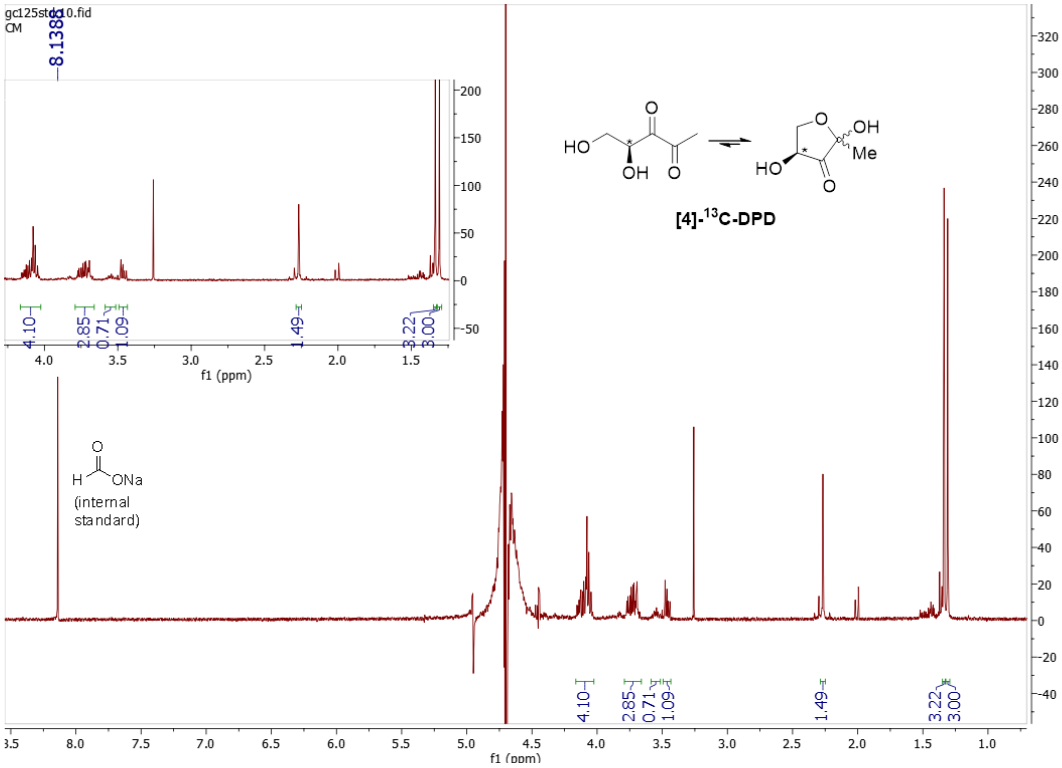

Figure S23. – ^{1}H-NMR spectrum of compound 4-[^{13}C]-DPD in D_2O/H_2O 2/8.

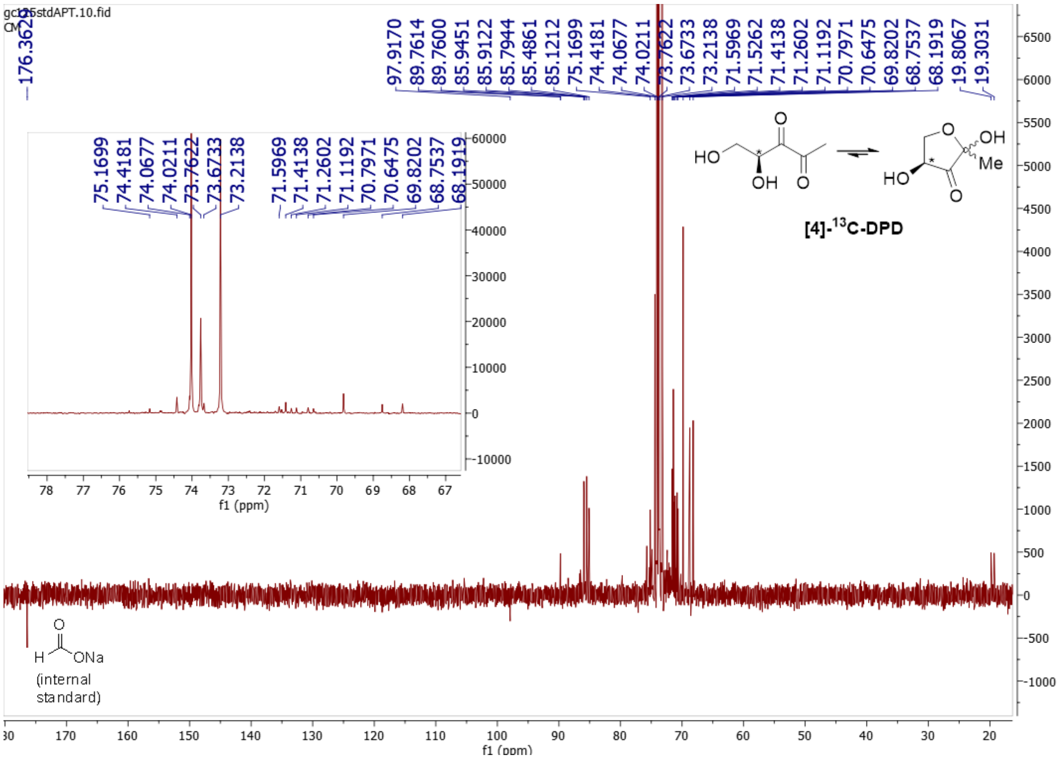

Figure S24. – ^{13}C-NMR spectrum of compound 4-[^{13}C]-DPD in D_2O/H_2O 2/8.

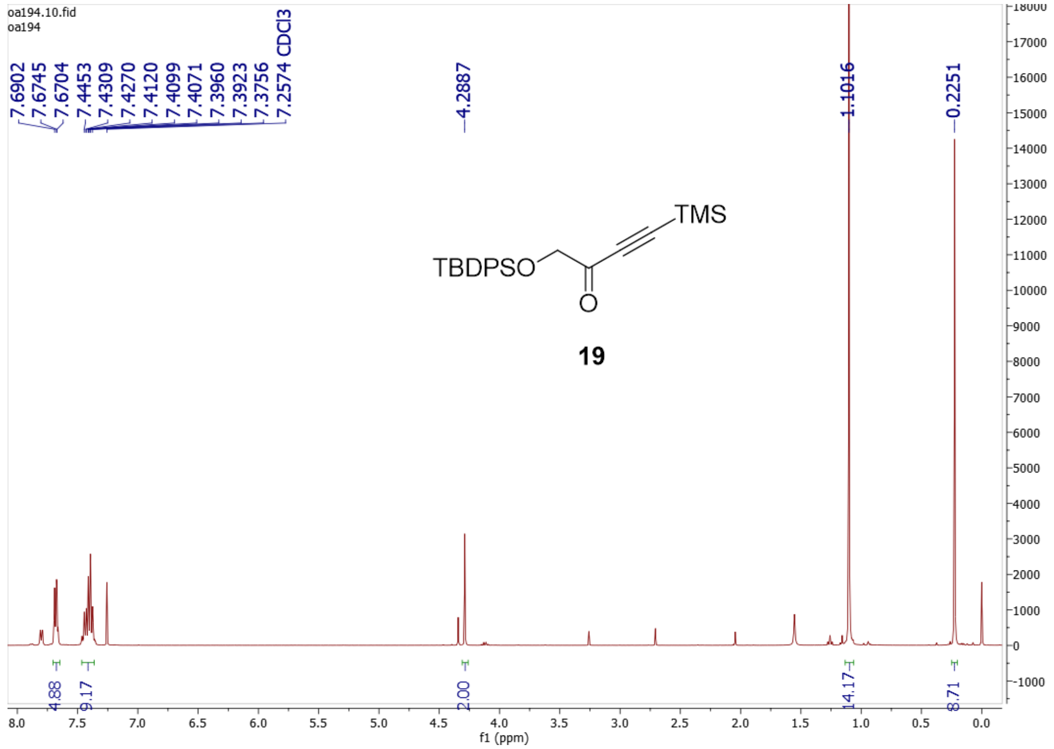

Figure S25. – ^{1}H-NMR spectrum of compound **19** in $CDCl_3$.

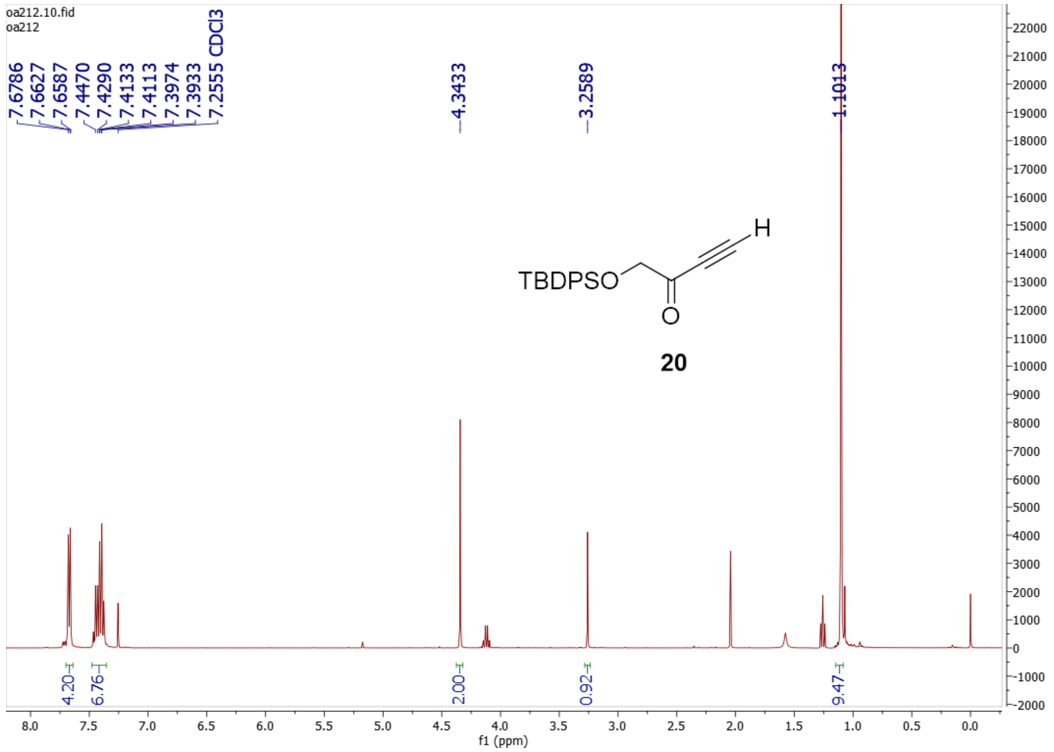

Figure S26. – ^{1}H-NMR spectrum of compound **20** in $CDCl_3$.

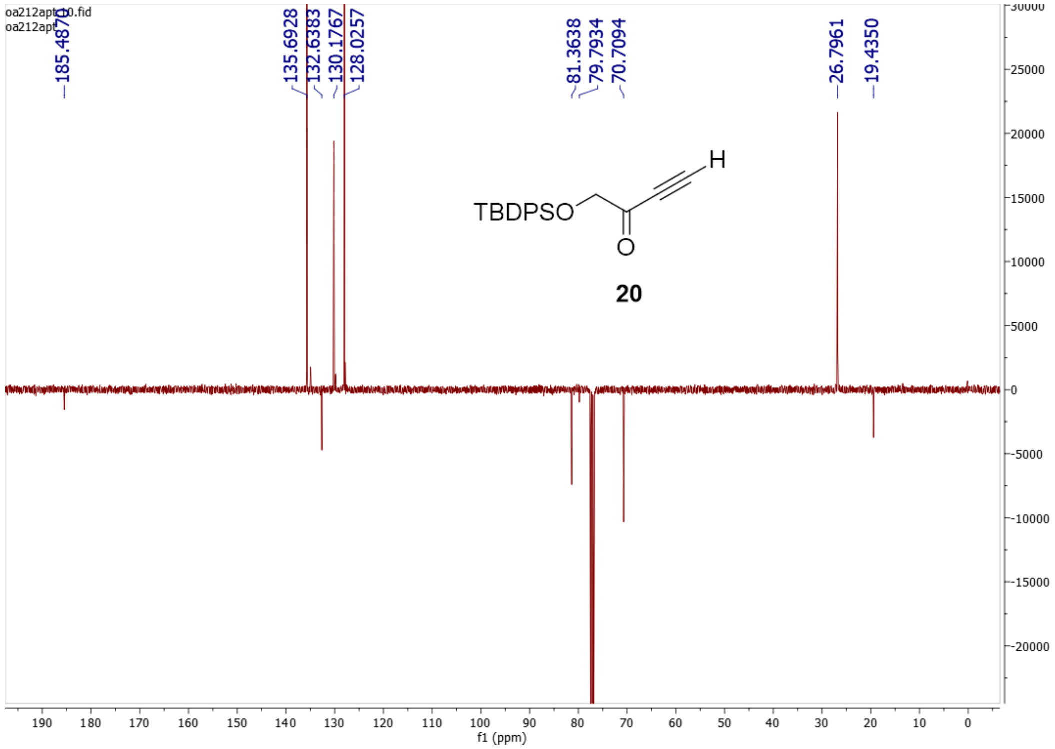

Figure S27. – APT spectrum of compound **20** in $CDCl_3$.

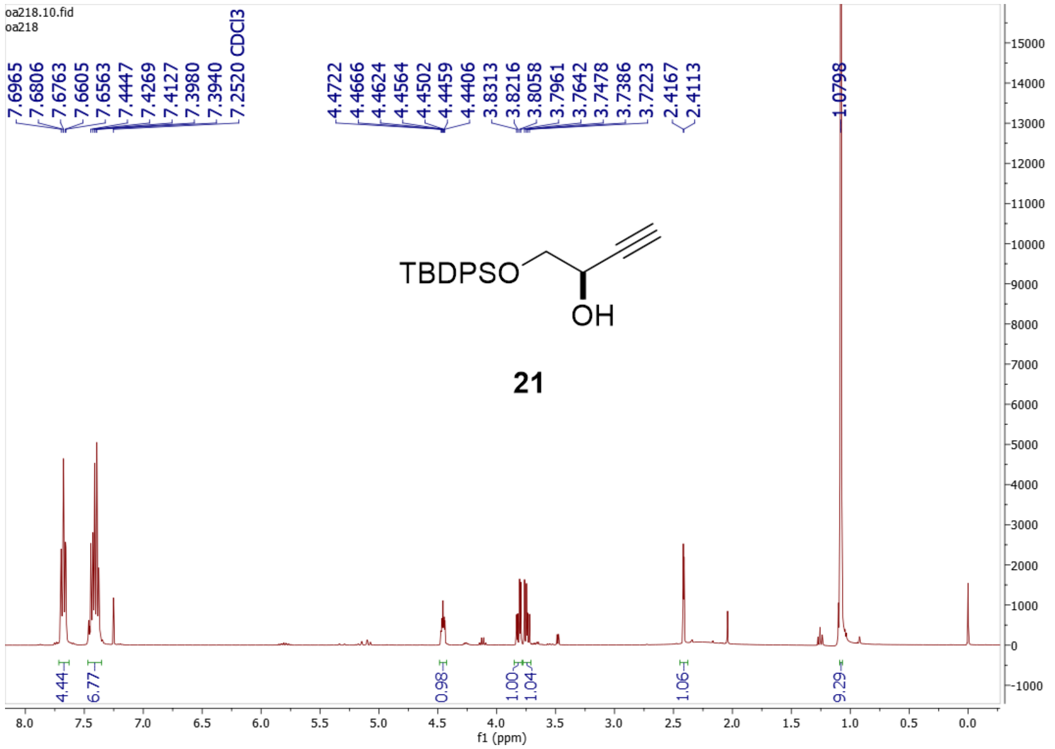

Figure S28. – ^{1}H-NMR spectrum of compound **21** in $CDCl_3$.

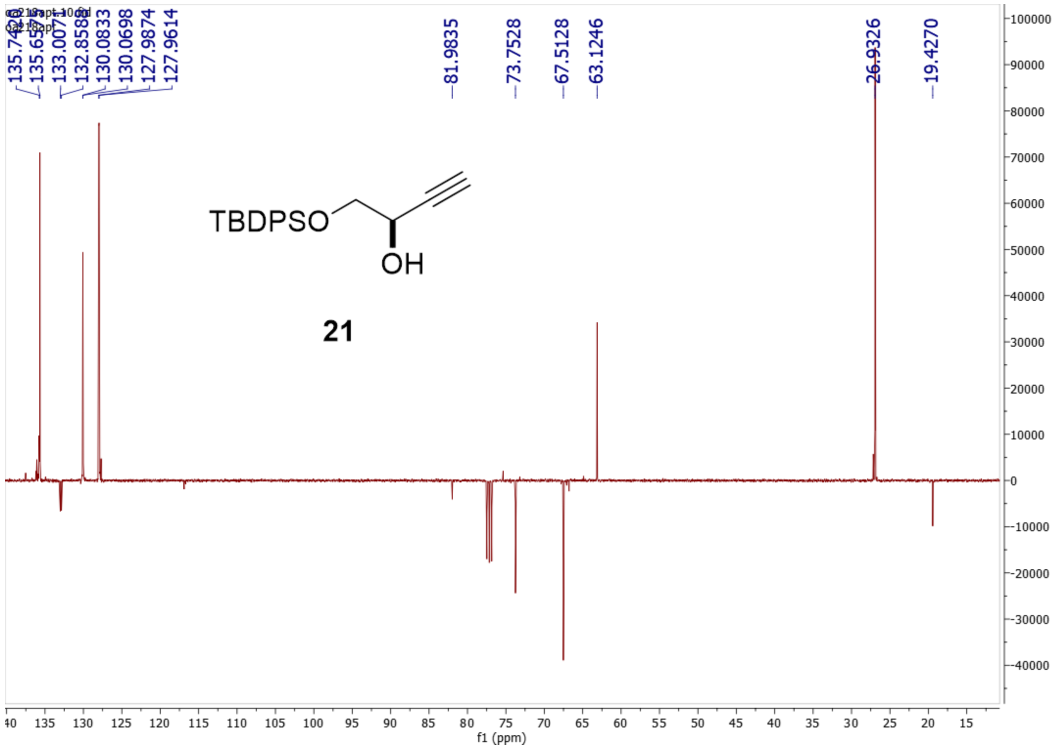

Figure S29. – APT spectrum of compound **21** in $CDCl_3$.

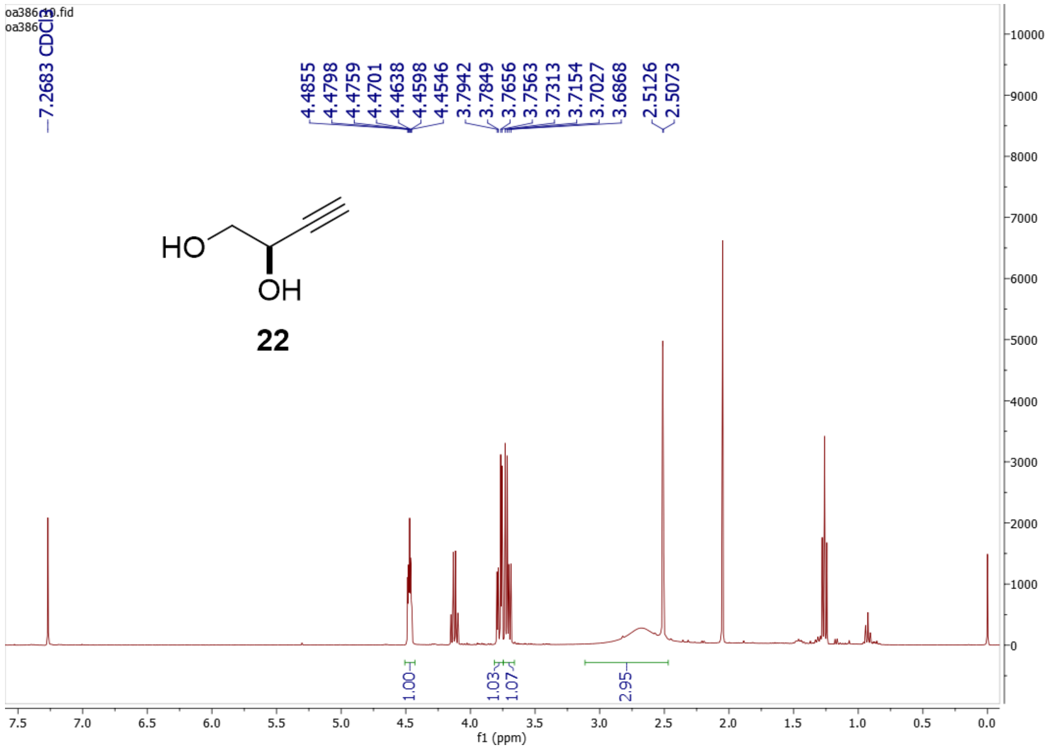

Figure S30. – ^{1}H-NMR spectrum of compound **22** in $CDCl_3$.

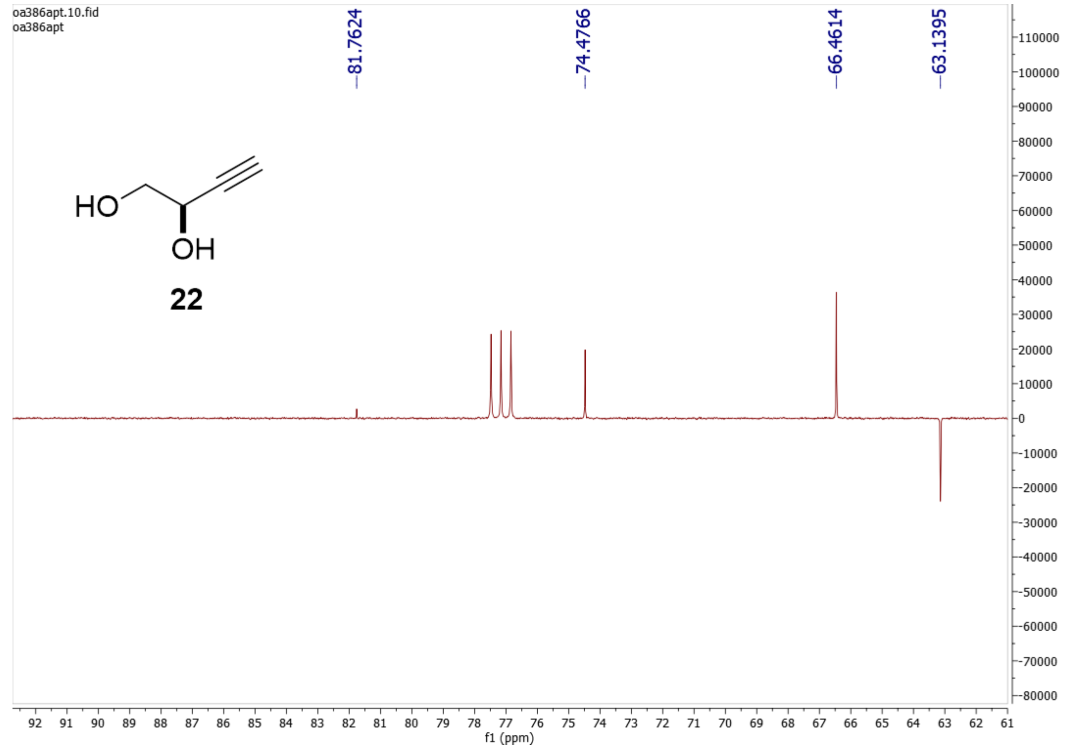

Figure S31. – APT spectrum of compound **22** in $CDCl_3$.

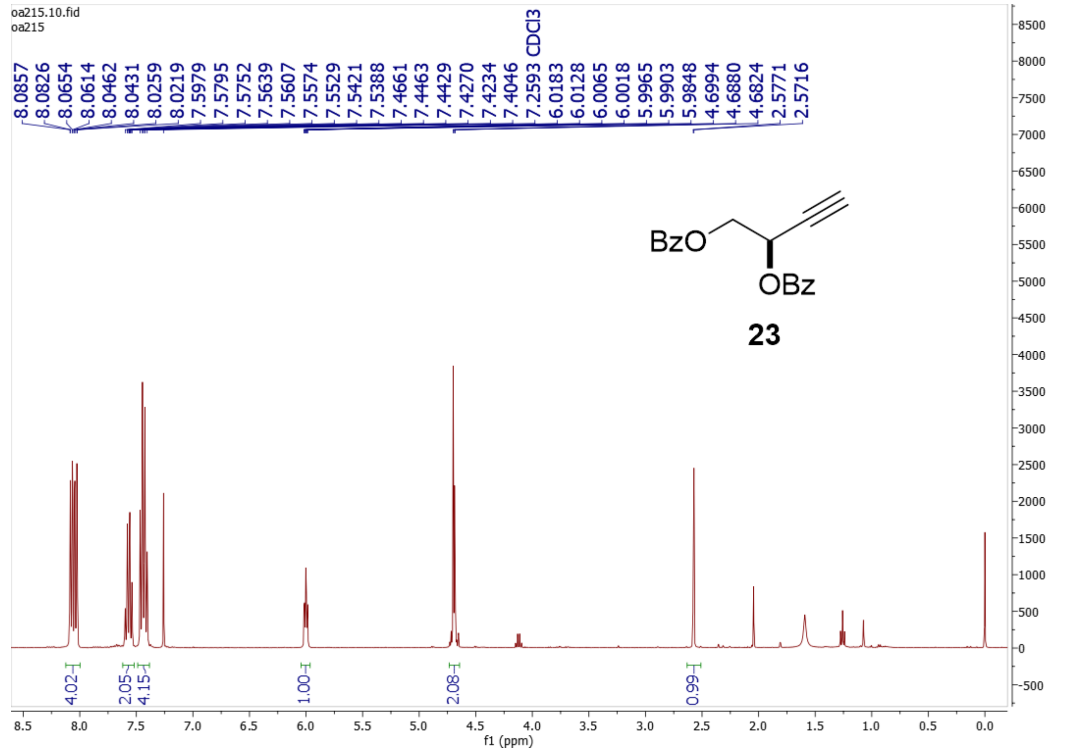

Figure S32. – ^{1}H-NMR spectrum of compound **23** in $CDCl_3$.

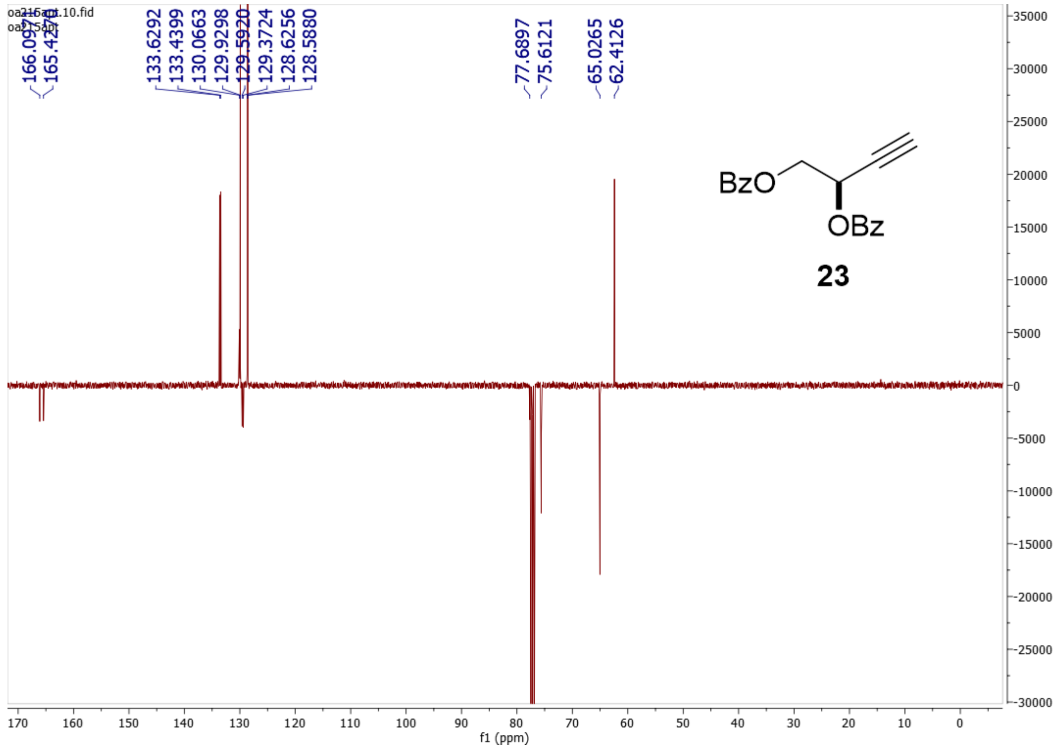

Figure S33. – APT spectrum of compound **23** in $CDCl_3$.

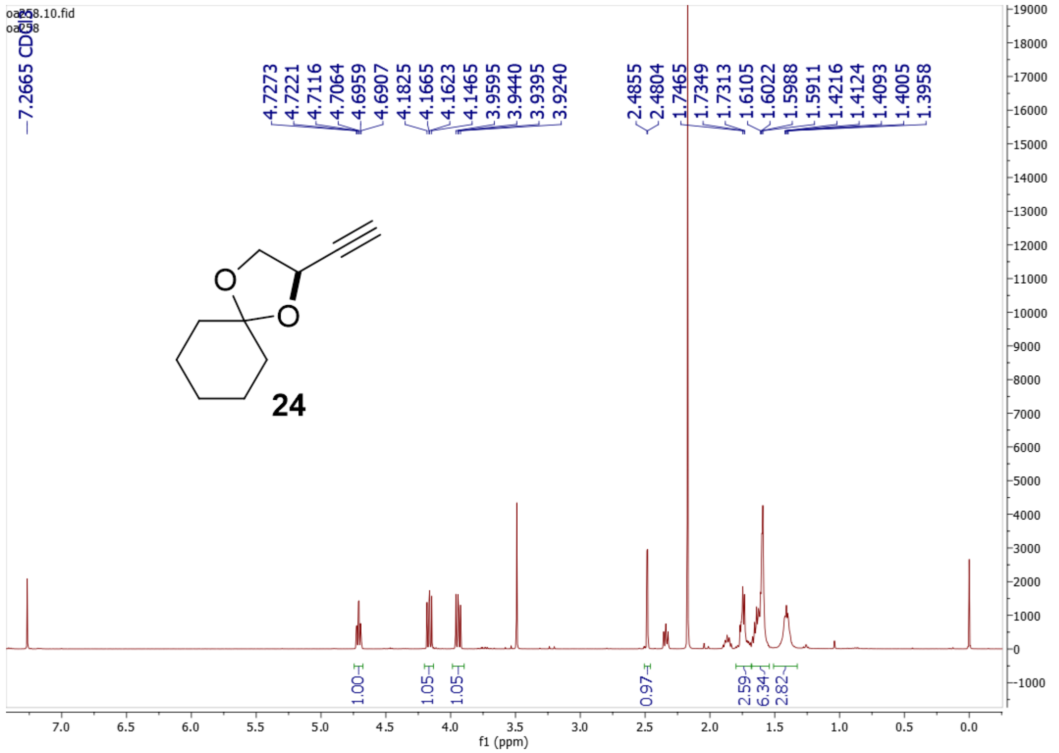

Figure S34. – ^{1}H-NMR spectrum of compound **24** in $CDCl_3$.

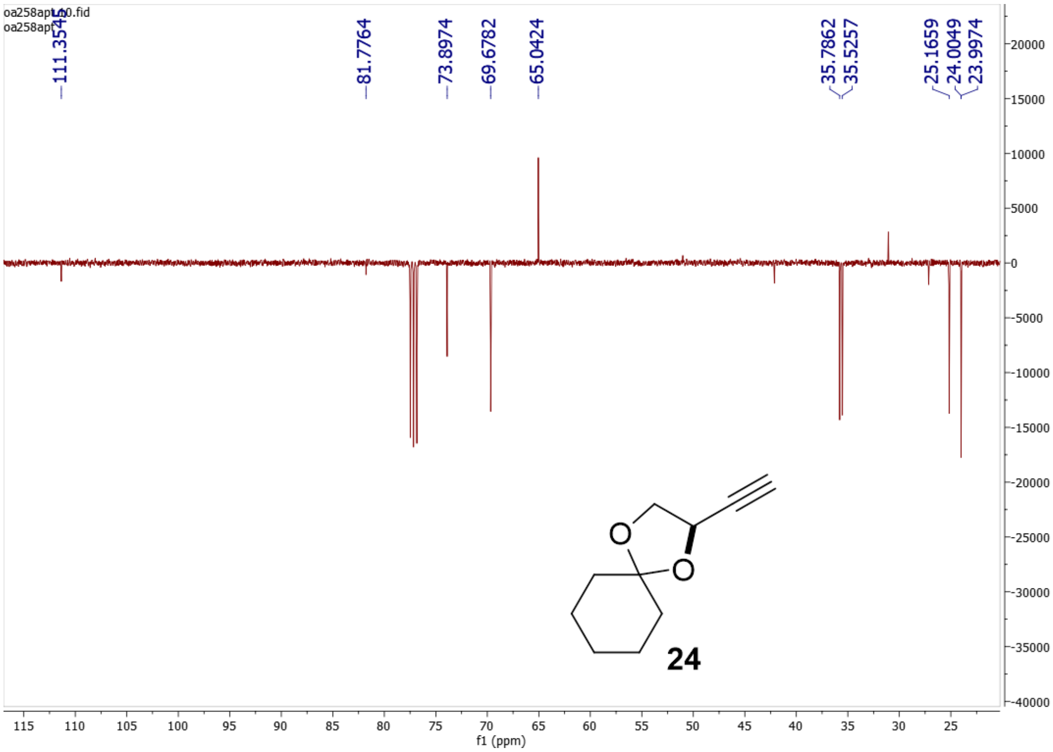

Figure S35. – APT spectrum of compound **24** in CDCl3.

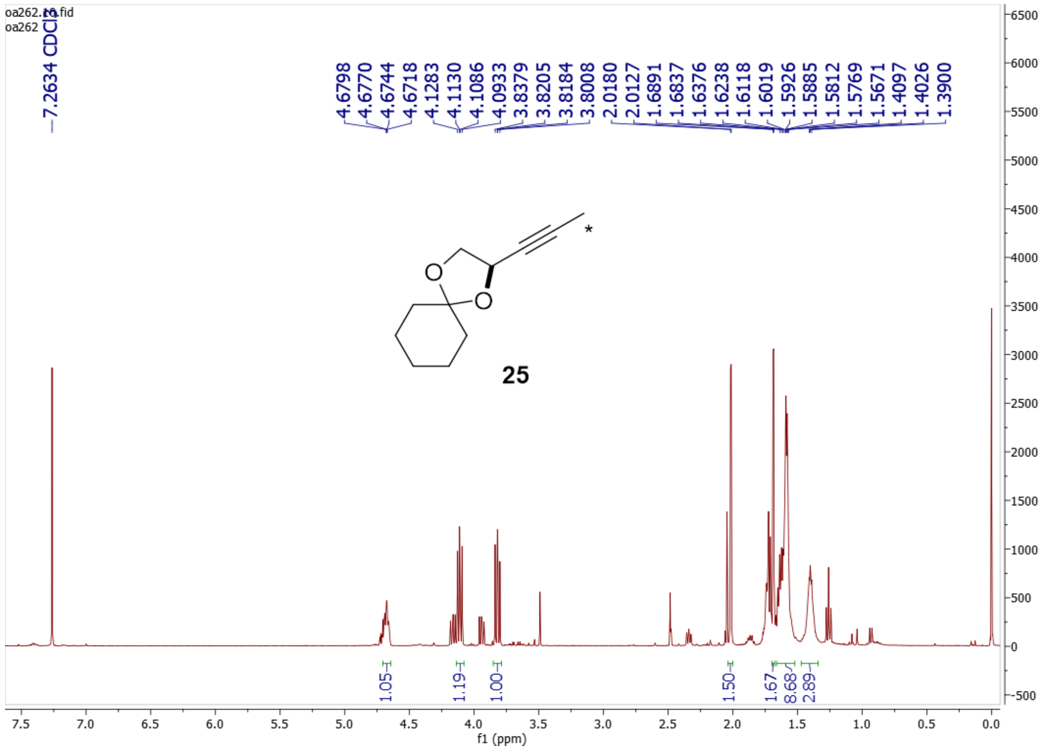

Figure S36. – ^{1}H-NMR spectrum of compound **25** in CDCl3.

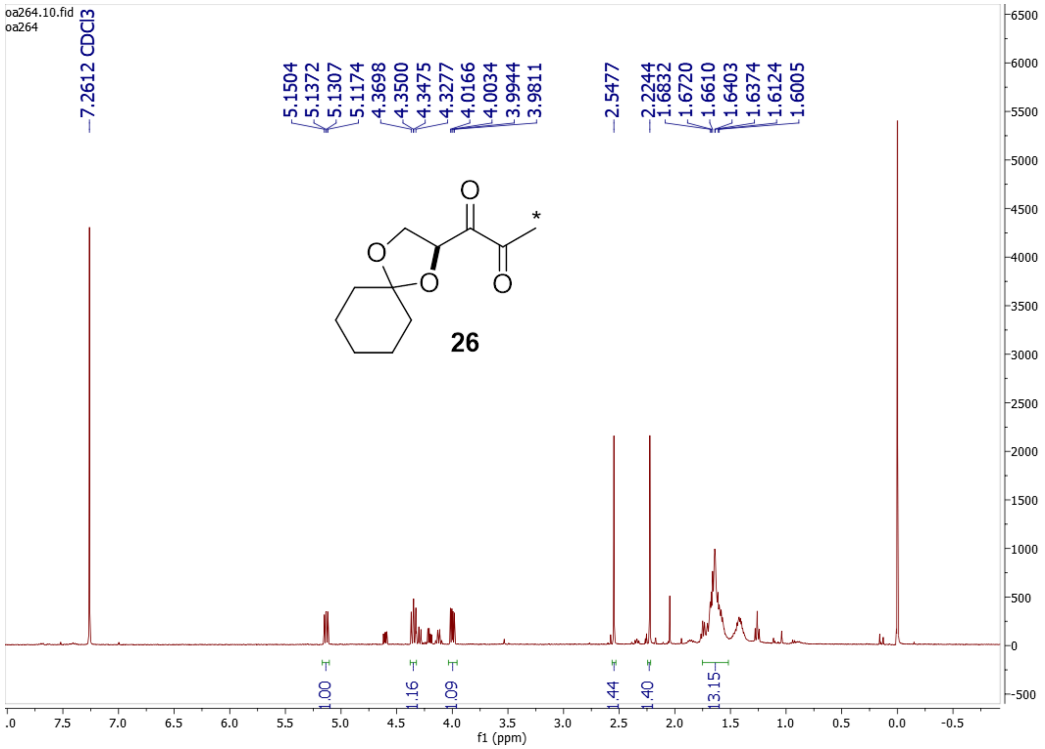

Figure S37. – ¹H-NMR spectrum of compound **26** in $CDCl_3$.

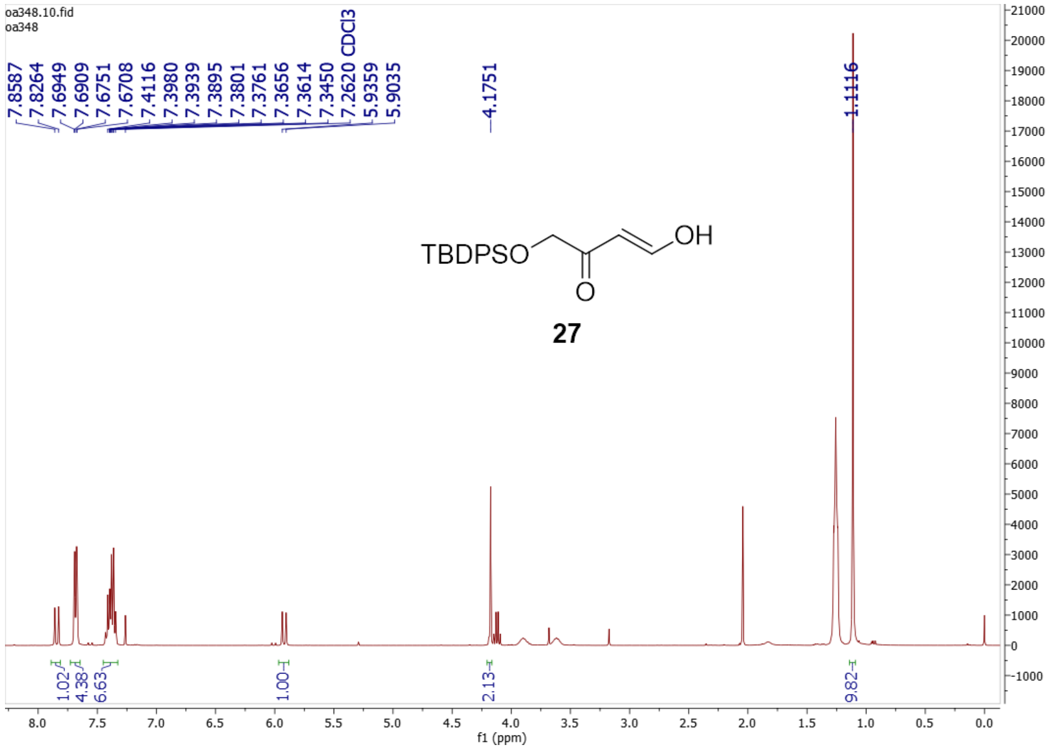

Figure S38. – ¹H-NMR spectrum of compound **27** in $CDCl_3$.

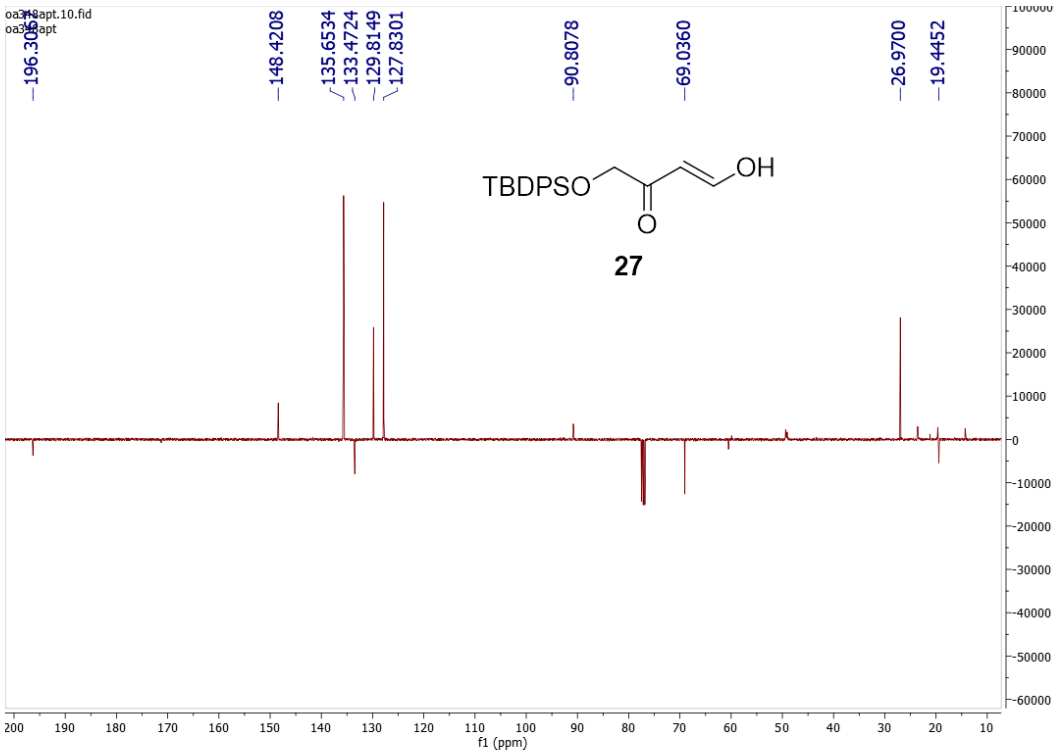

Figure S39. – APT spectrum of compound **27** in $CDCl_3$.
